# The *Gasdermin E* Gene Has Potential as a Pan-Cancer Biomarker, While Discriminating between Different Tumor Types

**DOI:** 10.3390/cancers11111810

**Published:** 2019-11-18

**Authors:** Joe Ibrahim, Ken Op de Beeck, Erik Fransen, Marc Peeters, Guy Van Camp

**Affiliations:** 1 Centre of Medical Genetics, University of Antwerp and Antwerp University Hospital, Prins Boudewijnlaan 43, 2650 Edegem, Belgium; joe.ibrahim@uantwerpen.be (J.I.); ken.opdebeeck@uantwerpen.be (K.O.d.B.); erik.fransen@uantwerpen.be (E.F.); 2 Centre for Oncological Research, University of Antwerp and Antwerp University Hospital, Wilrijkstraat 10, 2650 Edegem, Belgium; marc.peeters@uza.be; 3 StatUa Centre for Statistics, University of Antwerp, 2000 Antwerp, Belgium; 4 Department of Medical Oncology, Antwerp University Hospital, Wilrijkstraat 10, 2650 Edegem, Belgium

**Keywords:** DNA methylation, biomarker, pan-cancer, tumor-specific, Gasdermin E, detection

## Abstract

Due to the elevated rates of incidence and mortality of cancer, early and accurate detection is crucial for achieving optimal treatment. Molecular biomarkers remain important screening and detection tools, especially in light of novel blood-based assays. DNA methylation in cancer has been linked to tumorigenesis, but its value as a biomarker has not been fully explored. In this study, we have investigated the methylation patterns of the *Gasdermin E* gene across 14 different tumor types using The Cancer Genome Atlas (TCGA) methylation data (N = 6502). We were able to identify six CpG sites that could effectively distinguish tumors from normal samples in a pan-cancer setting (AUC = 0.86). This combination of pan-cancer biomarkers was validated in six independent datasets (AUC = 0.84–0.97). Moreover, we tested 74,613 different combinations of six CpG probes, where we identified tumor-specific signatures that could differentiate one tumor type versus all the others (AUC = 0.79–0.98). In all, methylation patterns exhibited great variation between cancer and normal tissues, but were also tumor specific. Our analyses highlight that a *Gasdermin E* methylation biomarker assay, not only has the potential for being a methylation-specific pan-cancer detection marker, but it also possesses the capacity to discriminate between different types of tumors.

## 1. Introduction

Cancer is the second leading cause of death worldwide with 9.6 million deaths and 17 million new cases occurring yearly [1]. The five most prevalent cancers worldwide, include lung, breast, colorectal, prostate, and gastric cancer [1]. Despite advances in diagnosis and treatment, the socio-economic burden of cancer still weighs heavily on societies worldwide. Novel, accurate, and cost-effective diagnostic strategies are needed for improved treatment and optimal disease management [2,3,4]. In recent years, the use of biologically identifiable characteristics, more commonly known as biomarkers, to indicate the presence of cancer in the body has gained considerable attention. Studies have examined several sources of biomarkers, including DNA mutations, metabolites, gene and protein expression, mRNA, imaging, and antibodies amongst others [5,6,7].

More recently, epigenetic alterations, most notably DNA methylation, have garnered much attention in the context of putative cancer markers for diagnosis and early detection [8,9,10,11,12,13]. In brief, DNA methylation is the addition of a methyl group predominantly to cytosine bases on the DNA backbone. Aberrant DNA methylation patterns are considered a hallmark of cancer [14]. Several studies have demonstrated the repression of tumor suppressor genes involved in cellular signalling pathways, via promoter hypermethylation. Global genomic hypomethylation has also been associated with genomic-instability and silenced gene re-expression [15,16,17]. Various studies have already outlined the potential of methylation as a biomarker for the early detection, diagnosis, and prognosis of cancer [12,13,18,19,20,21,22,23,24,25]. Only four commercially available DNA methylation analytical kits for cancer diagnosis currently exist. These use the genes *VIM* (cologuard) [26] and *SEPT9* (Epi proColon, ColoVantage and RealTime mS9) [27] for colorectal cancer, *SHOX2* (Epi prolong) in lung cancer [28], and *GSTP1/APC/RASSF1A* (ConfirmMDx) in prostate cancer [29,30,31]. These assays, however, demonstrate varying performance across tumor stages and are often ineffective at detecting residual disease. More recently, Cohen et al. developed a blood-based assay, CancerSEEK, that assess the levels of circulating proteins and mutations in cell-free DNA to detect eight common cancer types, with sensitivities ranging from 69% to 98% [32]. Biomarkers, that are used to diagnose pan-cancer tumors, are yet to be identified, however, their eventual discovery could offer huge advantages for early detection and optimal clinical follow-up.

Our lab has a long history with the *Gasdermin E* (*GSDME*) gene, which was originally identified as being implicated in an autosomal dominant form of hearing loss and named *Deafness Autosomal Dominant 5* (*DFNA5*) [33]. More recently, its function as a tumor suppressor, through the activation of programmed cell death, was revealed [34]. The epigenetics of *GSDME* have been studied in several contexts; some studies have examined its epigenetic silencing through methylation in gastric and colorectal tumors [35,36,37], while more recent studies by our laboratory have highlighted it as a potential methylation-based biomarker for breast and colorectal cancers [38,39,40]. Lately, interest in this gene has been rekindled by studies exploring the mechanisms by which it induces cell death, again highlighting its important role to cancer formation [41,42,43]. Based on the exceptional in-silico performance of *GSDME* methylation as a diagnostic/early detection marker in breast and colorectal cancers, we postulated that its methylation patterns could be ubiquitous across several cancer types, a characteristic that could be leveraged for use as a “pan-cancer” biomarker. We further hypothesize that *GSDME* may likely possess distinctive methylation patterns in different tumors. Our study aimed to analyze *GSDME* methylation patterns in the largest cancer patient dataset to date (N = 6502) using publicly available data from The Cancer Genome Atlas (TCGA). We thus aimed to assess the capacity of *GSDME* methylation patterns to serve as effective detection biomarkers in both a pan-cancer and tumor-specific context.

## 2. Results

### 2.1. GSDME Differential Methylation Across 14 Tumor Types

**Figure 1:**
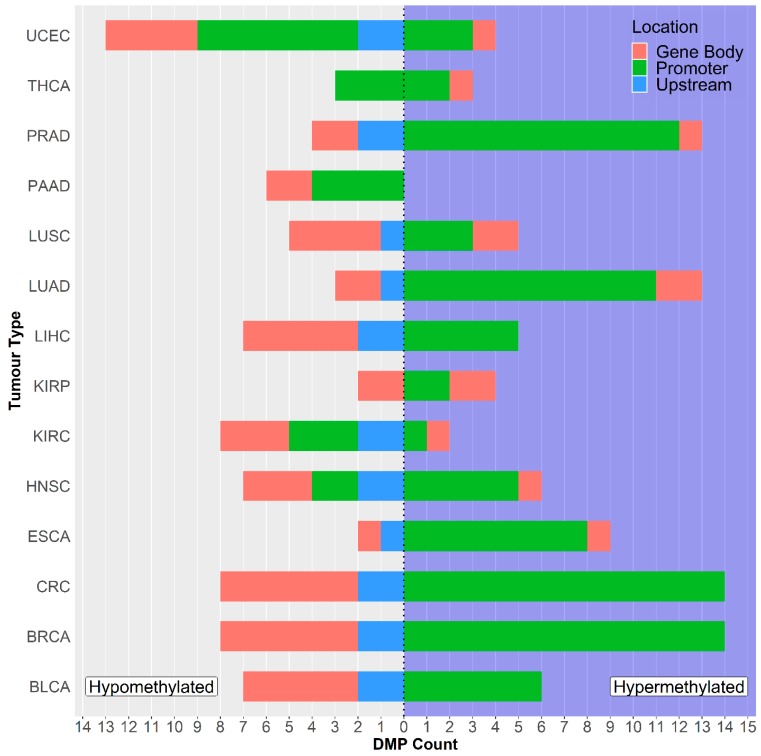
Countplot showing the number of differentially methylated *Gasdermin E* (*GSDME*) probes across the datasets. The right panel corresponds to hypermethylated (DNA methylation beta values of tumor samples are significantly higher than that of normal samples) CpGs, while the left panel corresponds to hypomethylated (DNA methylation beta values of tumor are significantly lower than that of normal) CpGs. Please refer to Table 1 for tumor dataset abbreviations.

**Figure 2:**
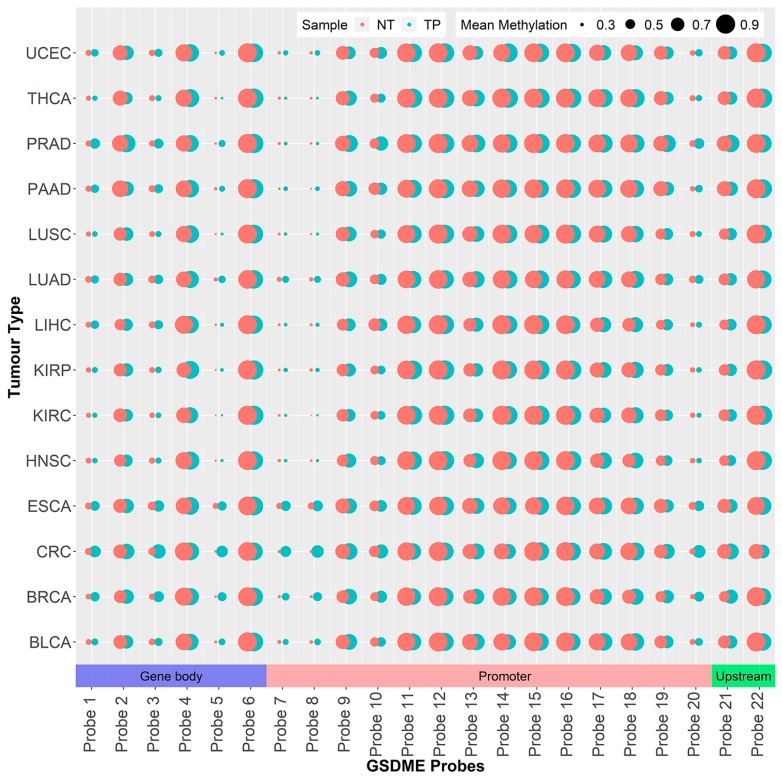
Map of the 22 *GSDME* GpGs showing the average probe methylation and chromosomal location across the different datasets. The size of the dots indicates the average methylation, while the colour indicates tissue type (NT = normal tissue, TP = tumor tissue). Please refer to Table 1 for tumor dataset abbreviations.

To comprehensively explore the methylation patterns of *GSDME*, we investigated differential methylation in 14 different tumors, by comparing cancer samples with corresponding normal tissue, at a distance from the tumor. Differential methylation was significantly variable amongst the different cancer types; on average 13 out of the 22 CpG probes were differentially methylated between tumor and normal tissues (*p* = 3.107 E-30 to 4.96 E-2) (Supplementary Table S1). No significant correlation was found between the number of differentially methylated probes and dataset sizes (Pearson’s correlation *p*-value > 0.05). In the breast and colorectal cancer datasets, all 22 *GSDME* CpGs were differentially methylated, while the kidney, pancreatic, and thyroid tumors exhibited differential methylation in only six CpGs (Figure 1 and Supplementary Table S1). In general, those differentially methylated probes were hypomethylated in the normal tissue, compared to the tumor tissues. Uterine carcinomas reported the highest count of hypomethylated *GSDME* CpGs, followed by breast, colorectal, and renal clear cell tumors, while breast and colorectal tumors, followed by lung and prostate tumors, had the highest count of hypermethylated CpGs (Figure 1 and Figure 2). Interestingly, differential methylation was not limited to promoter CpGs. In all of the tumor types investigated, one or more of the six intragenic probes were differentially methylated. Even probes in the region upstream of the promoter, which follow methylation patterns of gene body CpGs, were differentially methylated in 11 out of the 14 tumors (Figure 1 and Figure 2).

### 2.2. GSDME Methylation as a Pan-Cancer Detection Biomarker

#### 2.2.1. Initial Predictor Combination Selection

We used binary logistic regression to identify combinations of *GSDME* probes that could be used to differentiate tumors from normal samples across the different cancer types. In accordance with other studies on TCGA data [44,45], we only chose datasets that had a tumor-to-normal sample ratio of 10% or a minimum of ten tumor-normal pairs. Next, we pooled the 14 different tumor datasets, resulting in 719, and 5783 normal, and tumor samples, respectively. We regressed the binary models with combinations of one to six methylation probes as predictors and bootstrapped these calculations 1000 times each to avoid the case-to-control imbalance in the dataset. In total, 110,056 combinations were tested, of which 74,613 comprised six probes. The average area under the curve (AUC) was 0.627 using only a single probe, while 0.871 used a combination of six probes. Using combinations of seven or more probes, we encountered model overfitting with diminishing returns, considering the major increase in the number of combinations to test, with only minimal improvements in AUC. Single probes were less than optimal for discrimination between cases and controls, the best of which, probe 6, scored an AUC of 0.737, while the rest had AUCs in the 0.60s range. While relevant, these findings are unsurprising as information obtained from only one predictor is too little to make a clear distinctions, given the considerable heterogeneity of the samples and the inherent diversity between the different tumors. Another factor involved in these interpretations is the narrow dynamic range associated with the Beta-value, which only extends from 0 to 1, thus, limiting the size of discernible differences to one single position. In contrast, the models employing combinations of five to six probes, as predictors, performed exceptionally well across the cancer types, with AUCs reaching 0.862, and 0.871, respectively. The combination of probes with the best predictive power included probes 3, 12, 14, 18, 20, and 21. Of these probes, one is in the putative gene body region, four are in the promoter, and one is present in the upstream region (Figure 2 and Table 2). The top scoring combinations also included the mentioned probes in addition to the promoter probes 11, 13, and 19 in an array of combinations.

#### 2.2.2. Individual Dataset Analysis

**Figure 3:**
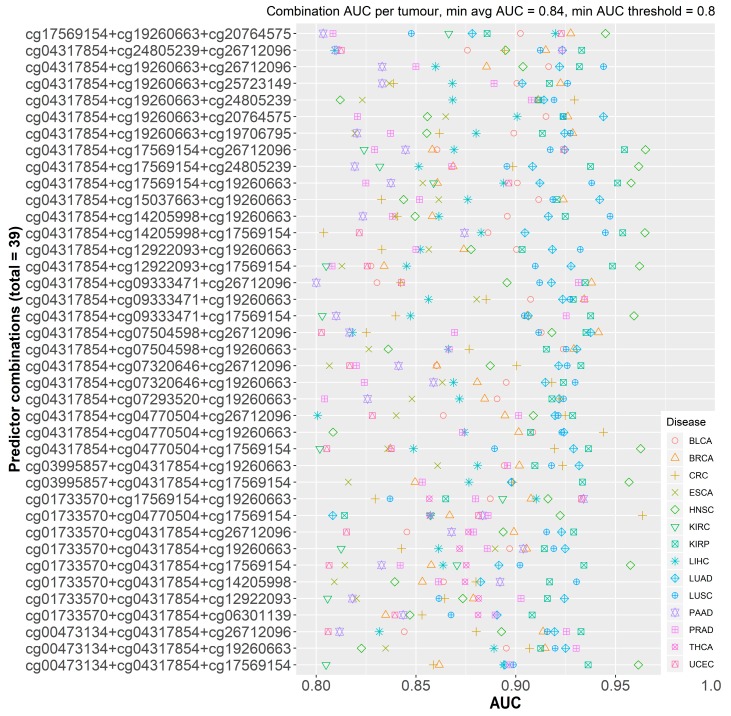
Cleveland plot of the calculated average area under the curves (AUCs for 39 probe combinations that satisfy both filters (minimum average AUC = 0.84 and minimum AUC threshold = 0.80) across the datasets.

**Figure 4:**
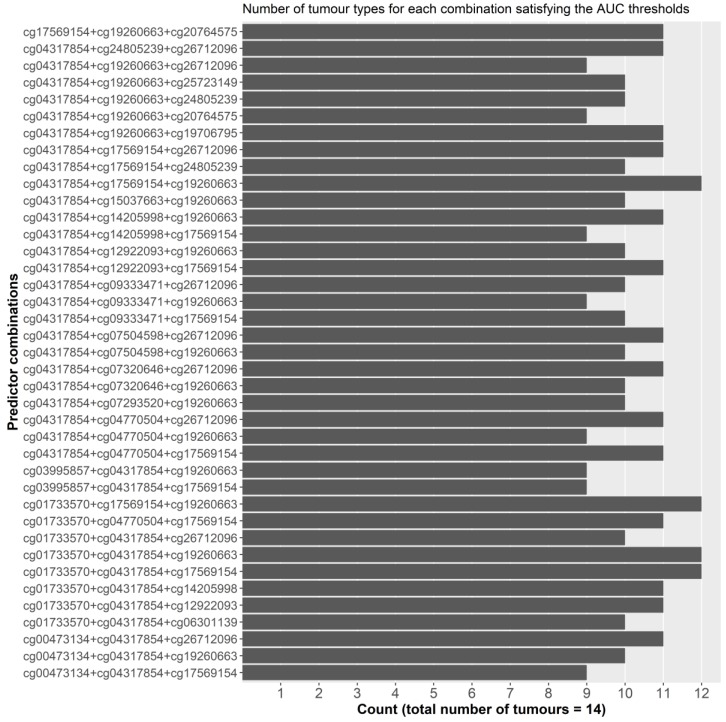
Countplot of the number of tumor types per combination that satisfy the AUC filters.

**Figure 5:**
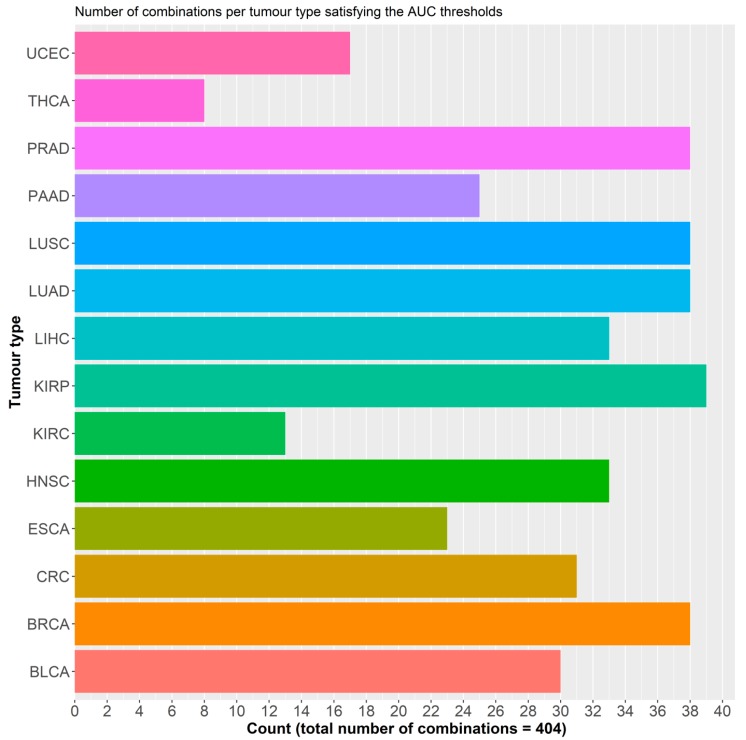
Countplot of the number probe combinations that satisfy the filters for each of the datasets. Please refer to Table 1 for tumor dataset abbreviations.

To ensure that dataset sample size did not cause any bias for the selection model in the pooled dataset, we then reproduced the same analysis in the 14 individual datasets separately. For these combinations to possess pan-cancer functionality, they must; (i) present consistently high AUCs across the different datasets with a relatively small standard deviation, and (ii) larger datasets should not be correlated with better AUCs. Single probes performed better, on average, in the individual datasets with an AUC of 0.810. This can be attributed to the smaller samples of these datasets and the decrease in heterogeneity amongst the two sample classes. A total of 1540 different combinations of three probes (more than three predictors resulted in model overfitting) were tested, with varying AUC outcomes, ranging from 0.520 to 0.974. No discernible effect of sample size on AUC was observed. In order to combine the results from both analyses and select the best performing probe combinations, we set two filters. For both the pooled and individual analyses, we set the minimum average AUC in bins of 0.1 increments, starting at 0.80 and ending at maximal AUC. Additionally, for the individual analysis, the minimum threshold for any probe combination should not be below 0.80 (Figure 3) (Supplementary Table S2). This resulted in 14 combinations, with an AUC of 0.85 or more, in the pooled analysis: in this scenario the top recurring probes to these combinations were probes 4, 6, and 16. In the individual analysis, 7 combinations fit the two filters and the top recurring probes to these combinations were probes 4, 14, and 16. Thirty nine combinations of 3 probes satisfied the 0.84 AUC filter with several demonstrating AUCs above 0.90 for breast, colorectal, prostate, kidney, and lung cancers (Figure 3), which are amongst the most common cancer types worldwide [1]. Four combinations that included probes 3, 5, 6, and 14 satisfied the set filters in 12 of the 14 tumor types, followed by 14 others in 11 of the 14 types (Figure 4). Kidney tumors, followed by pancreatic, prostate, lung, and breast had the highest number of probe combinations that satisfied the set filters at 39 and 38 combinations respectively (Figure 5).

#### 2.2.3. Final Model and Validation

**Figure 6:**
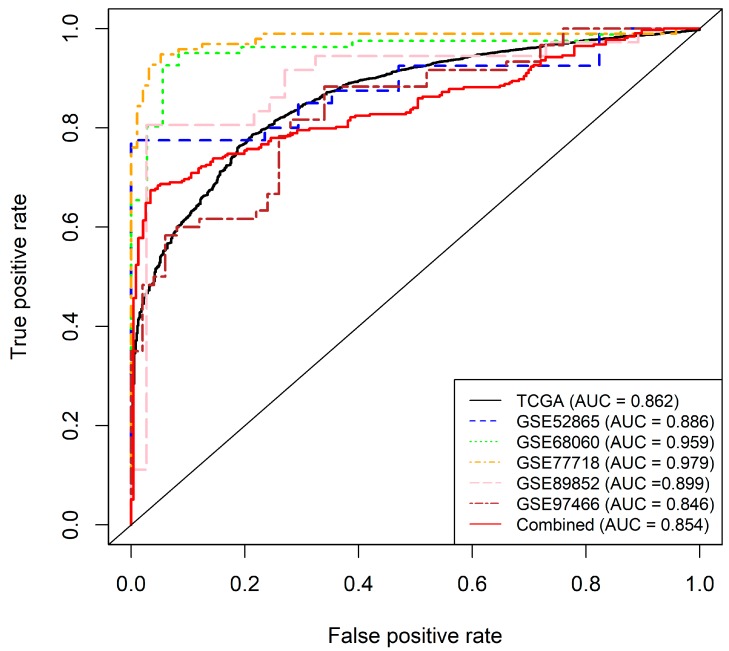
Receiver operating characteristic (ROC) curves for the final *GSDME* pan-cancer model along with the validation datasets. The black solid curve represents the training dataset, the red solid line represents the combined validation dataset, while the dotted lines represent the individual validation sets. The final model included six CpG probes; one in the gene body (Probe 3), four in the promoter region (Probes 12, 14, 18 and 20) and one in the upstream region (Probe 21) and accounted for age and tumor stage. Sensitivity and specificity at various cut-off values for the datasets are plotted. The final model yielded an AUC of 0.86 (95% CI: 0.852–0.87). At a set cut-off of 0.55, sensitivity and specificity were at 98.8%, and 93.2%, respectively, while overall model accuracy was 89.7%. The right panel shows ROC curves for the subsequent validation of the model by three external datasets. The diagonal line represents the line of no discrimination between tumor and normal tissues.

The top six probes from the pooled analysis (probes 3, 12, 14, 18, 20 and 21,) were then selected for further model construction and validation. A logistic regression model was implemented, based on the selected six features and trained on the pooled dataset, involving the 14 tumor types (N = 6502). This logistic regression model achieved a 10-fold cross validated AUC of 0.86 in the training set (Figure 6). Applying a 0.55 cut-off value, the sensitivity, specificity, and overall accuracy were 98.8%, 94.2%, and 89.7% respectively. We then independently validated the constructed model using five external datasets downloaded from the Gene Expression Omnibus (GEO) (GSE52865 breast cancer [46], GSE68060 colorectal cancer, GSE77718 colorectal cancer [47], GSE89852 hepatocellular cancer [48], GSE97466 thyroid cancer [48]), as well as a pooled dataset of the five. The AUCs for those five independent datasets were 0.89, 0.96, 0.97, 0.90, 0.85 respectively, and 0.85 for the pooled set (Figure 6). To assess the homogeneity of the relationship between CpG methylation and sample type, we included tissue type as a dependent variable, and added CpG methylation, stage, and the interaction between methylation and stage as independent variables in the logistic regression model. We then tested the significance of the interaction term using a likelihood ratio test, comparing the fit of the model with both, main effects and their interaction term against the model, with only the main effects of methylation and stage. We did not find any significant effects of disease stage or age on tissue type prediction, and thus, concluded that methylation was not significantly altered by stage. In all, the six-probe model demonstrated a good predictive power in a pan-cancer setting, and its consistent performance in external datasets shows its validity as a detection marker.

### 2.3. GSDME Methylation as a Tumor-Specific Biomarker

**Figure 7:**
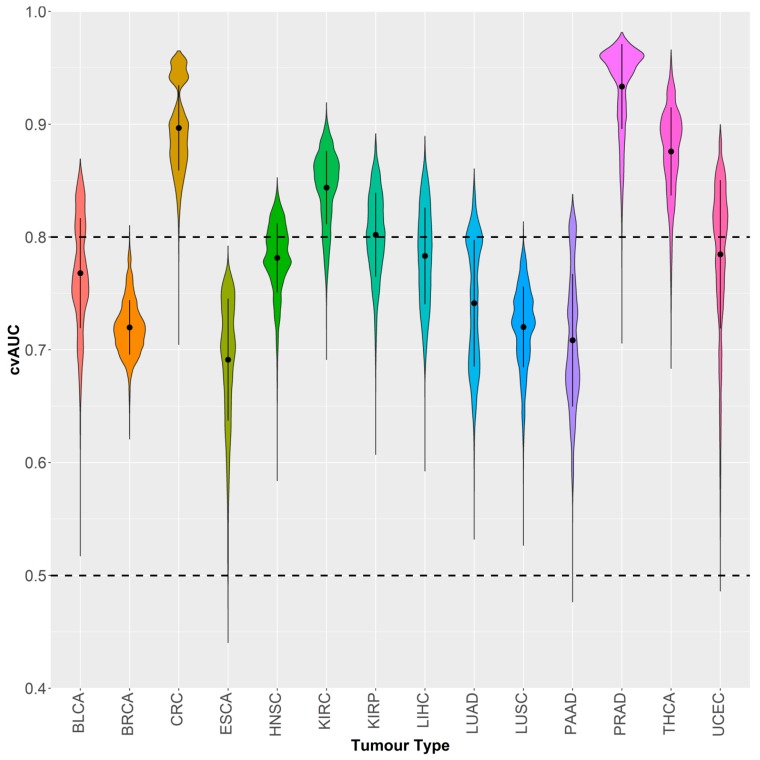
Violin plot of the distribution of partial least squares-discriminant analysis (PLSDA) cross-validated AUCs of different probe combinations (74,613) classifying each of the 14 tumor types against all others. Please refer to Table 1 for tumor dataset abbreviations.

**Figure 8:**
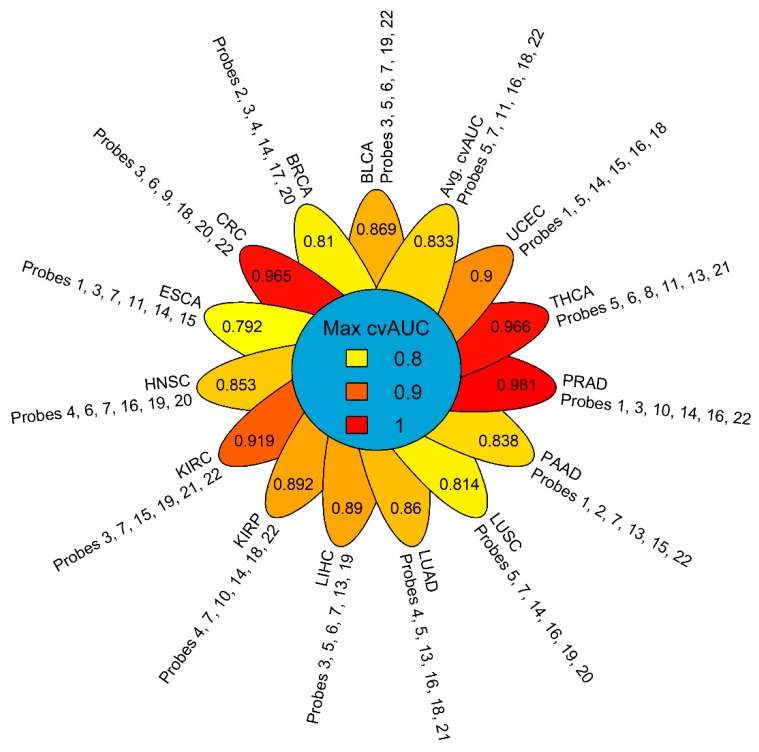
Flower plot of the maximum calculated cross-validated AUC for classifying each of the 14 tumors against all others, along with the corresponding probe combination that yielded the displayed AUC. Please refer to Table 1 for tumor dataset abbreviations.

We explored the capacity of *GSDME* methylation to differentiate between different tumor types based on the combinations of CpG probes. We again decided on the combinations of six probes, as preliminary testing showed the highest average AUC, with the least number of predictors, and the most reasonable number of combinations to test. We used the Partial Least Squares Discriminant Analysis (PLSDA) to fit models for 74,613 combinations using a pooled dataset of tumors across the 14 types (N = 5783). PLSDA is well-suited for multi-class predictive modelling, works well with large datasets and has demonstrated merit in medical diagnostics [49,50,51,52]. The average cross-validated AUC for classifying the 14 tumor types was 0.833 and was achieved using probes 5, 7, 11, 16,18 and 22. A large portion of combinations performed well in detecting colorectal, kidney, prostate and thyroid tumors with local AUC means above the 0.80 mark (Figure 7 and Figure 8). Other tumor types showed a wider spread in AUCs with lower means; however, the local AUC maxima were all 0.80 or above (Figure 7). Prostate cancer could be discriminated with the highest power against all other tumors (AUC = 0.981) followed by thyroid (AUC = 0.966), colorectal (AUC = 0.965) and kidney (AUC = 0.919) cancers. Esophageal tumors were the most problematical to discriminate amongst the tumor types with an AUC of 0.792, which is still acceptable in a prediction setting (Figure 8). The best performing combinations for all the predictions, included probes 3, 5, 7, 14, 19, and 22, which comprised all three regions of the *GSDME* gene, and were not limited to the promoter region where the greatest variations in methylation would typically be expected.

### 2.4. The Relation of GSDME Methylation to RNA-seq Expression and Clinicopathological Parameters

We examined *GSDME* expression levels, using RNA-seq data, downloaded from TCGA. The mean expression in normal tissues was 7.99, while it was slightly lower in tumor tissues at 7.80, but these differences were not significant. In general, higher expression levels could be observed in the normal tissues, as compared to the tumors. The only exception were head and neck, kidney, esophageal, lung, and liver tumors (Supplementary Table S3 and Supplementary Figure S1). Contrary to the general dogma, we could not find a very significant effect of methylation on RNA-seq expression in *GSDME*. On average the methylation of 5 of the 22 probes was significantly associated with RNA-seq expression, and the methylation of 3 probes, on average, per tumor type showed an association with gene expression. The head and neck, as well as kidney renal papillary carcinomas, showed a significant association between the methylation of 9 *GSDME* probes with gene expression. Whereas, pancreatic cancer showed an association in only 2 probes. Probe 22 exhibited associations across the most tumor types (10 types), while probe 19 did not show any association between methylation and expression levels in any of the cancer types. In general, the significant associations had negative slopes indicating an inverse relationship between methylation and expression. However, these slopes were not very large, hence, their true effect is still questionable. Moreover, there is no clear association between *GSDME* expression and methylation as these relations were not ubiquitously significant across promoter or gene body probes, in the majority of tumor types (Supplementary Table S4). We also analyzed the effect of clinicopathological parameters, namely age at diagnosis, gender, and ethnicity, on the methylation of GSDME, using linear models. Although, some of the *p*-values were lower than the significant p-value, their corresponding slopes were almost at 0, hence, their effect on methylation is negligible (Supplementary Table S5).

## 3. Discussion

This is the first study of its kind to explore *GSDME* methylation in multiple cancer types. In accordance with previous findings, differential methylation was observed for all 22 probes in breast cancer [39,40,53] and colorectal cancer [36,37,38] (Figure 1). Overall, differential methylation is observed mainly in promoter CpGs, which is not unexpected, given the nature of epigenetic modifications and the fact that a larger portion of the Illumina array probes is located in the *GSDME* promoter region and not the gene body. This might be different for newer arrays, such as the Illumina EPIC, which interrogates 50 *GSDME* CpGs, 20 of which are in the gene body region. In the mentioned promoter probes, hypermethylation is mainly reported, which is in agreement with the general epigenetic model of tumor suppressor genes in cancer [14,54]. Differential methylation was also observed in gene body CpGs (Figure 1 and Figure 2 and Supplementary Table S1). This is in line with studies that have described differential methylation in shore regions (lower CpG density regions that lie within 2 kilo base pairs up and downstream of a CpG island) genome-wide [55,56,57], and with the widespread hypomethylation, observed in cancer genomes, and which leads to disease progression by altering chromatin structure and stability [56]. To date, the precise function of gene body CpGs in the cancer landscape is yet to be defined. Several potential roles have been postulated, including modulating alternative promoters and long-range regulation [58,59], failsafe against spurious transcription initiation, and safeguarding against cryptic transcription initiation by RNA polymerase II [60]. Cancer is generally characterised by widespread genomic hypomethylation, particularly in gene body, shore, and intergenic regions. This ultimately highlights the complex role of functional epigenetic signalling, and reinforces the importance of such regions in tumor propagation, in light of aberrant methylation. Overall, it seems that differentially methylated probes, including the ones that are not located in the putative promoter, could play a key role in tumorigenesis and are valuable in the context of tumor classification.

In a diagnostic setting, only a handful of methylation biomarkers have made their way to the clinic, and no well-established pan-cancer biomarkers exist today [8]. Although, the current tests offer a much improved specificity over existing methods, they still show a variability linked to the tumor stage [8,61]. Recently, two separate studies highlighted the potential in DNA methylation biomarkers for cancer diagnosis, but these were tested in only one [62], and four cancer types [63], respectively, hence their findings may not be strictly translatable to other cancers. By using a combination of six probes, our model could effectively classify tumor and normal tissues with high accuracy in a pan-cancer setting. Our external validation reinforces the model’s validity over external cohorts and its generalizability over a multitude of tissue types (Figure 6). CpGs, in all three genomic regions, were used for the final model and in several high performing predictor combinations. This highlights their contribution to tissue type predictions and the potential for upcoming technologies that can interrogate additional CpGs outside promoter regions and could yield more insight into their perturbed methylation patterns. Aberrant DNA methylation in cancer varies greatly, depending on the tumor stage, and although, biomarkers are still lacking in detecting early stage disease [22,64,65], our model was able to successfully predict tissue type in a large cohort of mixed stage samples. This could be very effective for use as an early detection marker [9].

One of the paramount practices in blood-based cancer detection is the clear identification of the tumor type and efficient tracking of its origin. To that end, we explored whether *GSDME* methylation profiles could be used as predictors for tumor type. We were able to identify the signatures of six probes that could accurately identify 14 tumor types (Figure 7 and Figure 8). Again, in tumor-specific settings, the most discriminatory signatures comprised the probes of all three genomic regions, contrary to the widespread focus on abnormal methylation of CpGs islands in gene promoters [16,52,53,54,55]. In both the pan-cancer and tumor specific analyses, gene body CpGs were part of the final model, indicating their importance in cancer. While, genomic variations may be responsible for a large percentage of the variability in DNA methylation in cancer tissue, a sizeable fraction of the aberrant DNA methylation in cancer may also arise from tissue-specific idiosyncratic pathogenic signalling cascades. [57]. Moreover, tissue specific methylation signatures have already been demonstrated for several tumor types [58,59,60,61,62,63]. The heterogeneity in the TCGA datasets adds another level of complexity to the predictions. The use of methylation was reliable for detecting disease-specific signatures and for employing them for classification. Our probe-based tumor-specific classifier was able to efficiently discern the different tumor types, thereby indicating that GSDME methylation is a potentially viable tumor-specific biomarker. Determining which tumor type the patient has, and identifying the origin of a tumor for metastatic patients, greatly impacts their treatment strategies [9,49]. For some primary tumors, biopsies are required for diagnosis and this can be hampered by the patient’s endurance of surgery or by the inaccessibility of tumor locations. Diagnosis is often still problematic, even after high-quality biopsies are obtained, and molecular identification is increasingly employed to better characterise a patient’s tumor [64,65,66].

The performance of *GSDME* methylation, in both pan-cancer and tumor-specific biomarker settings, makes the gene an attractive target for a minimally-invasive diagnostic/detection biomarker assay development. This can be done through array or digital droplet PCR technologies, that are applied to a blood-based assay. The only blood test currently used for early cancer diagnosis is the prostate-specific antigen assay. However, its use and efficacy are still contended [28]. For diagnosis, blood-based assays should have high specificity to avoid false positives and their entailing follow-up procedures and anxiety. Liquid biopsies, based on somatic mutations, have already shown high specificity by targeting driver gene mutations present exclusively in tumor tissues [66,67,68,69]. To date, however, these biopsies have only been evaluated on small patient cohorts and on patients with advanced-stage tumors. Their real specificity is, hence, still debateable when used in a diagnostic settings [70]. Our *GSDME* methylation-based model has shown very high sensitivity in silico and follows a conservative approach for classifying tissues, while minimising false positives. Using methylation data confers a significant advantage over mutations in liquid biopsies with respect to diagnostic sensitivity, as studies suggest that early stage cancer patients have less mutations than the detection limit of downstream mutation assessment technologies [69,71]. The very high sensitivity reached in our models is advantageous in both, diagnostic settings and is independent of tumor stage. Mutation based liquid biopsies alone, are not suitable for identifying tumor types as the same gene mutations can drive multiple tumor types. Recently, tumor specific methylation signatures have attracted significant interest for the nuanced identification of tumors [72,73,74]. Based on our findings, the unique combinations of methylation probes can be used to accurately differentiate tumor types and overcome the limitations of mutation analysis. Another aspect that can be leveraged for diagnosis, is the integration of DNA methylation with mutation information, protein levels, and expression data [75]. The CancerSEEK assay has already laid the foundations for such an approach by combining mutation data from 16 genes with eight protein biomarkers detectable in the plasma. Despite their highly-reported specificity, the authors argue that their approach could be further combined with other markers, including methylation, to increase sensitivity and localization of tumor site. Although, the CancerSEEK model is based on liquid biopsies, one of its shortcomings is that it has not yet been validated in independent cohorts, [32] our analysis, however, has exhibited comparable results in both training and validation datasets. Based on our analysis, *GSDME* methylation possesses significant potential as a highly discriminative biomarker within a multi-analyte test.

## 4. Materials and Methods

### 4.1. Datasets and Study Population

Level 3, 450K DNA methylation data and RNAseq V2 gene expression data were downloaded from the TCGA Data Portal (https://tcga-data.nci.nih.gov) using an in-house developed Python (version 2.7) script as described in Ibrahim et al. [38]. Although, TCGA houses data for more than 30 different tumors, some of the datasets had too few normal tissues for valid statistical analysis. We chose datasets that have a minimum case to control the ratio of 10%, and those with at least 10 control samples. In total, the datasets for 15 distinct tumors were downloaded. Colon and rectal tumor datasets were combined to form the colorectal cancer dataset, resulting in 14 unique datasets, the details of which are presented in Table 1. Similarly, biospecimen and clinical data files for the different datasets, were also downloaded. The samples in TCGA datasets were flash frozen/formalin-fixed paraffin-embedded, resection tissue samples, containing a minimum of 60% tumor nuclei and derived from primary, untreated tumor tissue.

Methylation values were obtained by TCGA using the Illumina Infinium HumanMethylation450 BeadChip microarrays (Illumina Inc., San Diego, California). Methylation is reported as β-value, which is the ratio of the methylated probe intensity over the sum of methylated and unmethylated probe intensities, ranging from 0 to 1. The Illumina 450K array includes 22 probes for the *GSDME* CpG sites, 16 of which are in the putative promoter, four are located in the putative gene body, while the remaining two are located in a region upstream of the putative promoter [38], the details of which are described in Table 2. A scheme showing the *GSDME* gene structure and CpG distribution can be found in Croes et al. [39] and Ibrahim et al. [38].

### 4.2. Statistical Analyses

We designated the following clinicopathological parameters from the TCGA clinical patient data files with which to perform association analyses: Age at diagnosis, gender, ethnicity, and pathological tumor stage (I–IV). The statistical software R (version 3.5.2) [76] was used to carry out all the statistical analyses. All reported p-values are two-sided, and those less than, or equal to, 0.05 were considered statistically significant. To account for the non-independence between measurements from the same individuals, a linear mixed model was fitted and included a random effect for the sample barcodes, while the significance of the fixed effects was tested via the F-test with a Kenward-Roger correction for the number of degrees of freedom. In all regression models, age was accounted for as a covariate, but it was excluded from the final model if its effect on the outcome was not significant. The relation between *GSDME* methylation and RNA-seq expression was examined using linear regression models, the analysis of variance and Spearman’s. The associations between methylation and the designated clinicopathological parameters were studied in a similar manner. In all regression models, age was accounted for as a covariate, but it was excluded from the final model if its effect on the outcome was not significant.

To assess the viability of *GSDME* methylation as a pan-cancer biomarker a two-fold approach was considered. In a first step, the analysis was carried out on the individual datasets. Binary logistic regression models were fitted to predict tissue type (normal/tumor) using different combinations of CpG methylation values as predictors. Stepwise multiple regression was used to determine the best combination of the 22 CpGs. The final model was chosen based on the highest Akaike information criterion (AIC) values with the lowest number of predictors possible. The accuracy of the model predictions was assessed by plotting receiver operating characteristic (ROC) curves. A ten-fold cross validation of these results was then performed. In a second step, we aggregated all the different datasets into one large cohort comprising 719 normal and 5783 tumor samples. We then carried out a similar analysis to the one described above. This analysis, however, was bootstrapped 1000 times, each time considering an equal number of cases and controls (700) at random to prevent class imbalance artefacts and ensure model robustness. To test the potential of *GSDME* methylation as a tumor-specific biomarker, we used the partial least squares-discriminant analysis (PLSDA) algorithm to distinguish between the different cancers. To that end, all 14 datasets were pooled together, resulting in a pooled dataset of 5783 tumors each being 1 of 14 cancer types. The algorithm was run using combinations of six probes and ROC curves with AUC values were generated for predicting each cancer type against the 13 others. Moreover, additional Illumina 450K CpG methylation datasets were downloaded from the Gene Expression Omnibus (GEO) database (https://www.ncbi.nlm.nih.gov/geo/) (GEO accession numbers GSE52865 breast cancer [46], GSE68060 colorectal cancer, GSE77718 colorectal cancer [47], GSE89852 hepatocellular cancer [48], GSE97466 thyroid cancer [48]), and were used for the subsequent external validation. The final model was refit on each of the external datasets and the AUC was recalculated for the new predictions. The R packages, used in the analysis, can be found in the supplementary material.

## 5. Conclusions

We previously reported that *GSDME* methylation is a potential biomarker for the diagnosis of breast [39,40] and colorectal cancers [38]. Given our results presented here, we have strong indications that this gene holds even more potential in both, a pan-cancer and tumor specific detection setting. Differential *GSDME* methylation was studied for the first time, across 14 tumor types. We identified six CpG probes whose methylation signatures are effective at distinguishing different tumors from normal samples. Based on the exceptional in-silico performance and the fact that predictions were unaffected by tumor stage, *GSDME* methylation as a biomarker may be useful in the early detection setting, where tumor types are unknown, a priori, and heterogeneous. Taken together, these findings demonstrate the utility of methylation biomarkers for the molecular characterization of cancer, with implications for diagnosis and prognosis. *GSDME* has been proven to be a candidate gene for the development of a minimally-invasive blood-based assay, or integration in a gene panel of a multi-analyte test. Such tests would be more cost-effective and cheaper than screening tests for single cancers. Evidently, the next step would be to test our findings in collected tissue biopsies, from a mixed cohort of cancer patients and healthy individuals, followed by testing in liquid biopsies for example. Eight of the 14 cancer types analyzed here accounted for around 5.3 million deaths worldwide in 2018 [77], and their earlier detection could credibly improve patient outcome and reduce deaths.

## Figures and Tables

**Table 1:** Overview of the The Cancer Genome Atlas (TCGA) datasets used for the analysis.

Dataset Name (TCGA Abbreviation)	Number of NT	Number of TP	Total
Bladder urothelial carcinoma (BLCA)	21	418	439
Breast carcinoma (BRCA)	96	791	887
Esophageal carcinoma (ESCA)	16	185	201
Head and Neck squamous cell carcinoma (HNSC)	50	528	578
Kidney renal clear cell carcinoma (KIRC)	160	324	484
Kidney renal papillary cell carcinoma (KIRP)	45	275	320
Liver hepatocellular carcinoma (LIHC)	50	377	427
Lung adenocarcinoma (LUAD)	32	473	505
Lung squamous cell carcinoma (LUSC)	42	370	412
Pancreatic adenocarcinoma (PAAD)	10	184	194
Prostate adenocarcinoma (PRAD)	50	502	552
Thyroid carcinoma (THCA)	56	507	563
Uterine Corpus Endometrial Carcinoma (UCEC)	46	438	484
Colorectal carcinoma (CRC)	45	411	456
Total	719	5783	6502

NT = Normal tissue, TP = Primary tumor.

**Table 2:** Table outlining the *GSDME* Illumina Infinium HumanMethylation450 probes along with their genomic locations.

Probe Abbreviation	Probe Name (Illumina)	Genomic Coordinate *	Location	Chromosome
**Probe 1**	CpG17790129	24738572	Gene body	7
**Probe 2**	CpG14205998	24748668	Gene body	7
**Probe 3**	CpG04317854	24762562	Gene body	7
**Probe 4**	CpG12922093	24767644	Gene body	7
**Probe 5**	CpG17569154	24781545	Gene body	7
**Probe 6**	CpG19260663	24791121	Gene body	7
**Probe 7**	CpG09333471	24796355	Putative Promoter	7
**Probe 8**	CpG00473134	24796494	Putative Promoter	7
**Probe 9**	CpG03995857	24796553	Putative Promoter	7
**Probe 10**	CpG07320646	24796981	Putative Promoter	7
**Probe 11**	CpG07293520	24797192	Putative Promoter	7
**Probe 12**	CpG04770504	24797363	Putative Promoter	7
**Probe 13**	CpG24805239	24797486	Putative Promoter	7
**Probe 14**	CpG01733570	24797656	Putative Promoter	7
**Probe 15**	CpG25723149	24797680	Putative Promoter	7
**Probe 16**	CpG22804000	24797691	Putative Promoter	7
**Probe 17**	CpG07504598	24797786	Putative Promoter	7
**Probe 18**	CpG15037663	24797835	Putative Promoter	7
**Probe 19**	CpG19706795	24797839	Putative Promoter	7
**Probe 20**	CpG20764575	24797884	Putative Promoter	7
**Probe 21**	CpG06301139	24798175	Upstream Region	7
**Probe 22**	CpG26712096	24798855	Upstream Region	7

* Location on genome build GRCh37/hg19.

## Supplementary Materials

The following are available online at https://www.mdpi.com/2072-6694/11/11/1810/s1, Figure S1: Interaction plot for the RNA-seq expression data showing the differences in expression levels between tumour and normal samples across the different tumour types. Table S1: Differences in GSDME CpG methylation (β-value) between normal and tumour tissue samples across the 14 different cancer types; Table S2: Table of the individual dataset analysis AUC values that satisfy the specified thresholds (minimum average AUC = 0.84 and minimum AUC threshold = 0.80). NA values are tumour types for which the probe combination did not meet the AUC thresholds; Table S3: Summary of the RNA-seq expression levels in the different sample types and across the different tumour types; Table S4: Table of the linear regression results for the analysis of RNA-seq expression and methylation; Table S5: Table of the linear regression results for the analysis of age and methylation.
