# Supplementary material for: The Gasdermin E Gene Has Potential as a Pan-Cancer Biomarker, While Discriminating between Different Tumor Types
Source: Cancers (Basel). 2019 Nov 18;11(11):1810. doi: 10.3390/cancers11111810 (PMC6896019; doi:10.3390/cancers11111810)
Supplement: Supplementary file 1 [file cancers-11-01810-s001.pdf]

cancers MDPI

The *Gasdermin E* gene Potential as a Pan-Cancer Biomarker, While Discriminating between Different Tumor Types

Joe Ibrahim, Ken Op de Beeck, Erik Fransen, Marc Peeters and Guy Van Camp

Table S1. Differences in GSDME CpG methylation (β-value) between normal and tumour tissue samples across the 14 different cancer types.

Probe	Mean Diff	Se Diff	KR_p-value	Mean TP	SD mean TP	SEM TP	Mean NT	SD mean NT	SEM NT	Tumour Type
cg17790129	-1.19E-01	2.96E-02	3.31E-04*	7.63E-01	2.11E-01	1.03E-02	8.92E-01	2.29E-02	5.00E-03	BLCA
cg14205998	-7.37E-02	1.93E-02	6.77E-04*	8.10E-01	1.92E-01	9.42E-03	9.33E-01	1.32E-02	2.88E-03	BLCA
cg04317854	-3.01E-02	3.05E-02	3.36E-01	6.87E-01	1.96E-01	9.58E-03	7.21E-01	7.54E-02	1.65E-02	BLCA
cg12922093	-1.07E-01	2.85E-02	7.08E-04*	7.12E-01	1.98E-01	9.69E-03	8.49E-01	3.27E-02	7.13E-03	BLCA
cg17569154	-1.19E-01	3.87E-02	4.02E-03*	5.71E-01	2.24E-01	1.10E-02	7.45E-01	9.99E-02	2.18E-02	BLCA
cg19260663	-1.31E-01	2.94E-02	8.91E-05*	7.38E-01	2.03E-01	9.92E-03	9.13E-01	2.95E-02	6.44E-03	BLCA
cg09333471	2.33E-03	1.93E-02	9.05E-01	2.10E-01	1.35E-01	6.58E-03	2.03E-01	5.29E-02	1.15E-02	BLCA
cg00473134	6.60E-03	1.18E-02	5.83E-01	1.06E-01	1.16E-01	5.66E-03	7.61E-02	3.05E-02	6.65E-03	BLCA
cg03995857	4.37E-02	2.05E-02	4.25E-02*	1.45E-01	1.51E-01	7.36E-03	9.67E-02	3.61E-02	7.87E-03	BLCA
cg07320646	4.39E-02	2.54E-02	9.58E-02	9.55E-02	1.86E-01	9.08E-03	3.31E-02	2.24E-02	4.89E-03	BLCA
cg07293520	1.63E-02	1.37E-02	2.48E-01	7.63E-02	1.36E-01	6.66E-03	3.83E-02	3.86E-02	8.42E-03	BLCA
cg04770504	5.16E-02	1.85E-02	9.37E-03*	9.72E-02	1.54E-01	7.54E-03	2.35E-02	1.15E-02	2.51E-03	BLCA
cg24805239	5.96E-02	2.22E-02	1.16E-02*	1.37E-01	1.46E-01	7.16E-03	7.32E-02	3.20E-02	6.99E-03	BLCA
cg01733570	-2.17E-02	2.76E-02	4.43E-01	4.38E-01	1.80E-01	8.79E-03	4.73E-01	5.34E-02	1.17E-02	BLCA
cg25723149	1.91E-02	3.16E-02	5.54E-01	4.14E-01	1.91E-01	9.34E-03	4.07E-01	5.97E-02	1.30E-02	BLCA
cg22804000	1.92E-03	2.85E-02	9.47E-01	3.88E-01	1.75E-01	8.55E-03	4.15E-01	4.61E-02	1.01E-02	BLCA
cg07504598	1.15E-01	2.92E-02	3.09E-04*	6.15E-01	1.62E-01	7.91E-03	5.17E-01	6.34E-02	1.38E-02	BLCA
cg15037663	6.80E-02	2.79E-02	2.05E-02*	6.75E-01	1.60E-01	7.81E-03	6.44E-01	5.12E-02	1.12E-02	BLCA

**cg19706795**	5.78E-02	2.09E-02	9.47E-03*	7.68E-01	1.34E-01	6.55E-03	7.29E-01	3.84E-02	8.39E-03	BLCA
**cg20764575**	4.65E-02	2.43E-02	6.66E-02	7.68E-01	1.26E-01	6.16E-03	7.20E-01	4.70E-02	1.03E-02	BLCA
**cg06301139**	-2.89E-02	1.05E-02	1.06E-02*	8.82E-01	1.13E-01	5.52E-03	9.40E-01	1.28E-02	2.80E-03	BLCA
**cg26712096**	-9.12E-02	2.30E-02	4.45E-04*	7.60E-01	2.02E-01	9.90E-03	9.14E-01	1.81E-02	3.94E-03	BLCA
**Probe**	**Mean Diff**	**Se Diff**	**KR_p-value**	**Mean TP**	**SD mean TP**	**SEM TP**	**Mean NT**	**SD mean NT**	**SEM NT**	**Tumour Type**
**cg17790129**	-1.18E-01	1.98E-02	1.47E-08*	7.62E-01	2.37E-01	8.43E-03	9.02E-01	3.88E-02	3.96E-03	BRCA
**cg14205998**	-1.27E-01	1.96E-02	5.72E-10*	7.90E-01	2.12E-01	7.54E-03	9.23E-01	2.11E-02	2.16E-03	BRCA
**cg04317854**	-8.05E-02	1.84E-02	1.98E-05*	7.42E-01	1.95E-01	6.92E-03	8.35E-01	5.91E-02	6.03E-03	BRCA
**cg12922093**	-1.86E-01	1.95E-02	2.65E-18*	6.73E-01	2.15E-01	7.64E-03	8.72E-01	3.74E-02	3.82E-03	BRCA
**cg17569154**	-1.69E-01	1.80E-02	2.00E-17*	6.85E-01	2.11E-01	7.52E-03	8.63E-01	6.18E-02	6.31E-03	BRCA
**cg19260663**	-1.75E-01	1.86E-02	2.69E-18*	7.41E-01	2.00E-01	7.10E-03	9.24E-01	2.12E-02	2.16E-03	BRCA
**cg09333471**	1.62E-01	1.53E-02	3.82E-22*	3.41E-01	1.62E-01	5.76E-03	1.86E-01	6.62E-02	6.75E-03	BRCA
**cg00473134**	1.32E-01	1.50E-02	9.90E-17*	2.01E-01	1.54E-01	5.49E-03	7.25E-02	2.91E-02	2.97E-03	BRCA
**cg03995857**	1.98E-01	2.07E-02	3.74E-19*	2.81E-01	2.15E-01	7.66E-03	8.41E-02	3.88E-02	3.96E-03	BRCA
**cg07320646**	1.57E-01	2.28E-02	5.50E-11*	1.85E-01	2.47E-01	8.77E-03	2.33E-02	1.57E-02	1.60E-03	BRCA
**cg07293520**	9.66E-02	1.44E-02	2.57E-10*	1.31E-01	1.73E-01	6.14E-03	2.68E-02	1.03E-02	1.05E-03	BRCA
**cg04770504**	1.54E-01	1.71E-02	5.75E-17*	1.82E-01	1.87E-01	6.66E-03	1.99E-02	1.27E-02	1.30E-03	BRCA
**cg24805239**	1.71E-01	1.69E-02	1.23E-20*	2.30E-01	1.80E-01	6.39E-03	5.85E-02	2.69E-02	2.74E-03	BRCA
**cg01733570**	1.09E-01	1.99E-02	1.10E-07*	5.06E-01	2.07E-01	7.38E-03	3.99E-01	1.01E-01	1.04E-02	BRCA
**cg25723149**	1.98E-01	1.98E-02	5.49E-20*	4.86E-01	2.12E-01	7.52E-03	2.84E-01	9.26E-02	9.45E-03	BRCA
**cg22804000**	1.46E-01	1.82E-02	4.88E-14*	4.32E-01	1.98E-01	7.03E-03	2.83E-01	6.03E-02	6.16E-03	BRCA
**cg07504598**	1.99E-01	1.72E-02	1.03E-24*	6.00E-01	1.90E-01	6.75E-03	3.98E-01	7.74E-02	7.90E-03	BRCA
**cg15037663**	1.35E-01	1.91E-02	1.22E-11*	6.02E-01	1.99E-01	7.09E-03	4.72E-01	7.67E-02	7.83E-03	BRCA
**cg19706795**	1.08E-01	1.77E-02	3.21E-09*	6.66E-01	1.84E-01	6.53E-03	5.61E-01	6.99E-02	7.14E-03	BRCA

**cg20764575**	1.73E-01	1.75E-02	1.97E-20*	6.61E-01	1.80E-01	6.39E-03	4.87E-01	6.54E-02	6.68E-03	BRCA
**cg06301139**	-6.64E-02	1.41E-02	4.47E-06*	8.41E-01	1.53E-01	5.45E-03	9.10E-01	2.70E-02	2.75E-03	BRCA
**cg26712096**	-1.91E-01	2.06E-02	7.82E-18*	7.29E-01	2.21E-01	7.86E-03	9.26E-01	1.59E-02	1.62E-03	BRCA
**Probe**	**Mean Diff**	**Se Diff**	**KR_p-value**	**Mean TP**	**SD mean TP**	**SEM TP**	**Mean NT**	**SD mean NT**	**SEM NT**	**Tumour Type**
**cg17790129**	-9.19E-02	2.06E-02	1.94E-05*	8.30E-01	1.54E-01	7.62E-03	9.15E-01	2.38E-02	3.55E-03	CRC
**cg14205998**	-1.25E-01	2.09E-02	4.13E-08*	8.00E-01	1.74E-01	8.60E-03	9.28E-01	2.46E-02	3.66E-03	CRC
**cg04317854**	-4.26E-02	1.59E-02	9.06E-03*	7.92E-01	1.21E-01	5.97E-03	8.43E-01	4.75E-02	7.08E-03	CRC
**cg12922093**	-1.78E-01	2.04E-02	1.18E-13*	6.68E-01	1.80E-01	8.90E-03	8.41E-01	3.21E-02	4.79E-03	CRC
**cg17569154**	-8.54E-02	2.61E-02	1.48E-03*	5.54E-01	1.89E-01	9.32E-03	6.32E-01	1.03E-01	1.54E-02	CRC
**cg19260663**	-9.71E-02	2.00E-02	3.82E-06*	8.02E-01	1.51E-01	7.47E-03	9.00E-01	2.84E-02	4.23E-03	CRC
**cg09333471**	1.84E-01	2.33E-02	2.74E-12*	4.83E-01	1.90E-01	9.39E-03	3.09E-01	4.27E-02	6.36E-03	CRC
**cg00473134**	2.35E-01	2.22E-02	1.01E-17*	3.33E-01	1.95E-01	9.62E-03	1.15E-01	2.73E-02	4.07E-03	CRC
**cg03995857**	3.93E-01	2.72E-02	1.71E-25*	4.97E-01	2.41E-01	1.19E-02	1.40E-01	4.51E-02	6.73E-03	CRC
**cg07320646**	3.46E-01	3.98E-02	6.49E-15*	3.68E-01	2.94E-01	1.45E-02	2.41E-02	9.54E-03	1.42E-03	CRC
**cg07293520**	2.46E-01	2.97E-02	4.73E-13*	2.77E-01	2.46E-01	1.21E-02	2.72E-02	4.87E-03	7.26E-04	CRC
**cg04770504**	2.94E-01	2.50E-02	6.59E-22*	3.18E-01	1.94E-01	9.58E-03	2.10E-02	3.77E-03	5.62E-04	CRC
**cg24805239**	3.09E-01	2.80E-02	7.87E-20*	3.97E-01	2.22E-01	1.10E-02	8.64E-02	1.85E-02	2.75E-03	CRC
**cg01733570**	1.11E-01	2.81E-02	1.34E-04*	5.92E-01	2.09E-01	1.03E-02	4.82E-01	4.51E-02	6.73E-03	CRC
**cg25723149**	1.08E-01	2.62E-02	8.02E-05*	6.31E-01	1.99E-01	9.82E-03	5.27E-01	4.88E-02	7.27E-03	CRC
**cg22804000**	1.08E-01	2.58E-02	5.79E-05*	5.66E-01	1.95E-01	9.63E-03	4.57E-01	4.30E-02	6.41E-03	CRC
**cg07504598**	1.09E-01	2.16E-02	2.13E-06*	6.76E-01	1.73E-01	8.56E-03	5.66E-01	4.64E-02	6.92E-03	CRC
**cg15037663**	6.53E-02	2.47E-02	9.59E-03*	6.26E-01	1.72E-01	8.50E-03	5.57E-01	5.67E-02	8.45E-03	CRC
**cg19706795**	7.06E-02	1.97E-02	4.67E-04*	7.11E-01	1.34E-01	6.64E-03	6.38E-01	6.08E-02	9.07E-03	CRC
**cg20764575**	2.88E-02	1.38E-02	4.00E-02*	8.04E-01	9.26E-02	4.57E-03	7.75E-01	3.61E-02	5.38E-03	CRC

**cg06301139**	-9.22E-02	1.67E-02	3.30E-07*	8.04E-01	1.46E-01	7.19E-03	9.02E-01	3.36E-02	5.02E-03	CRC
**cg26712096**	-1.23E-01	2.76E-02	1.93E-05*	4.60E-01	2.01E-01	9.92E-03	5.72E-01	1.11E-01	1.65E-02	CRC
**Probe**	**Mean Diff**	**Se Diff**	**KR_p-value**	**Mean TP**	**SD mean TP**	**SEM TP**	**Mean NT**	**SD mean NT**	**SEM NT**	**Tumour Type**
**cg17790129**	3.07E-02	3.81E-02	4.46E-01	8.01E-01	1.53E-01	1.13E-02	7.48E-01	1.09E-01	2.72E-02	ESCA
**cg14205998**	-2.12E-02	2.39E-02	3.97E-01	8.69E-01	1.22E-01	8.95E-03	8.64E-01	1.67E-01	4.17E-02	ESCA
**cg04317854**	1.02E-01	3.13E-02	2.42E-03*	7.96E-01	1.23E-01	9.04E-03	6.94E-01	7.79E-02	1.95E-02	ESCA
**cg12922093**	-5.78E-02	2.89E-02	6.24E-02	7.84E-01	1.32E-01	9.70E-03	8.16E-01	1.07E-01	2.67E-02	ESCA
**cg17569154**	-1.03E-01	5.63E-02	8.57E-02	6.94E-01	2.28E-01	1.68E-02	7.51E-01	2.28E-01	5.71E-02	ESCA
**cg19260663**	-7.89E-02	2.63E-02	7.16E-03*	8.23E-01	1.29E-01	9.51E-03	8.75E-01	6.19E-02	1.55E-02	ESCA
**cg09333471**	1.35E-01	4.75E-02	1.01E-02*	3.26E-01	2.25E-01	1.65E-02	2.77E-01	1.77E-01	4.43E-02	ESCA
**cg00473134**	1.57E-01	5.09E-02	5.22E-03*	2.18E-01	2.25E-01	1.65E-02	1.07E-01	9.73E-02	2.43E-02	ESCA
**cg03995857**	2.25E-01	6.57E-02	2.20E-03*	3.02E-01	2.94E-01	2.16E-02	1.70E-01	2.22E-01	5.55E-02	ESCA
**cg07320646**	2.35E-01	7.70E-02	5.19E-03*	2.72E-01	3.23E-01	2.38E-02	1.09E-01	2.39E-01	5.99E-02	ESCA
**cg07293520**	2.18E-01	6.45E-02	2.28E-03*	2.42E-01	2.78E-01	2.05E-02	8.79E-02	1.75E-01	4.38E-02	ESCA
**cg04770504**	1.52E-01	5.38E-02	9.37E-03*	1.93E-01	2.23E-01	1.64E-02	8.36E-02	1.75E-01	4.38E-02	ESCA
**cg24805239**	1.78E-01	5.83E-02	5.43E-03*	2.50E-01	2.52E-01	1.86E-02	1.41E-01	1.86E-01	4.64E-02	ESCA
**cg01733570**	7.08E-02	5.09E-02	1.88E-01	5.04E-01	2.37E-01	1.74E-02	4.85E-01	1.29E-01	3.24E-02	ESCA
**cg25723149**	2.67E-02	5.05E-02	6.11E-01	4.44E-01	2.59E-01	1.90E-02	5.01E-01	1.65E-01	4.13E-02	ESCA
**cg22804000**	5.19E-03	4.71E-02	9.16E-01	4.19E-01	2.33E-01	1.71E-02	4.92E-01	1.43E-01	3.57E-02	ESCA
**cg07504598**	8.76E-02	3.40E-02	1.96E-02*	5.73E-01	1.93E-01	1.42E-02	5.56E-01	1.66E-01	4.15E-02	ESCA
**cg15037663**	2.22E-02	3.42E-02	5.34E-01	6.27E-01	1.75E-01	1.29E-02	6.26E-01	1.31E-01	3.26E-02	ESCA
**cg19706795**	1.79E-02	2.93E-02	5.58E-01	7.21E-01	1.47E-01	1.08E-02	7.12E-01	1.41E-01	3.52E-02	ESCA
**cg20764575**	2.72E-02	2.98E-02	3.81E-01	7.05E-01	1.60E-01	1.18E-02	7.13E-01	1.73E-01	4.32E-02	ESCA
**cg06301139**	-4.62E-02	1.51E-02	7.13E-03*	8.83E-01	1.00E-01	7.38E-03	8.94E-01	1.26E-01	3.16E-02	ESCA

**cg26712096**	-4.39E-02	4.87E-02	3.95E-01	7.94E-01	1.85E-01	1.36E-02	8.24E-01	2.32E-01	5.81E-02	ESCA
**Probe**	**Mean Diff**	**Se Diff**	**KR_p-value**	**Mean TP**	**SD mean TP**	**SEM TP**	**Mean NT**	**SD mean NT**	**SEM NT**	**Tumour Type**
**cg17790129**	-1.20E-03	1.06E-02	9.12E-01	8.89E-01	7.96E-02	3.46E-03	8.89E-01	4.39E-02	6.21E-03	HNSC
**cg14205998**	-9.46E-03	4.39E-03	3.57E-02*	9.18E-01	4.57E-02	1.99E-03	9.33E-01	1.37E-02	1.94E-03	HNSC
**cg04317854**	8.57E-02	1.27E-02	7.40E-11*	8.22E-01	8.71E-02	3.79E-03	7.37E-01	6.68E-02	9.45E-03	HNSC
**cg12922093**	-3.79E-02	9.97E-03	2.91E-04*	8.27E-01	8.51E-02	3.70E-03	8.70E-01	3.91E-02	5.53E-03	HNSC
**cg17569154**	-2.04E-01	2.55E-02	4.14E-14*	7.18E-01	1.82E-01	7.91E-03	9.20E-01	3.23E-02	4.56E-03	HNSC
**cg19260663**	3.53E-03	6.02E-03	5.63E-01	9.03E-01	5.60E-02	2.44E-03	9.03E-01	3.21E-02	4.54E-03	HNSC
**cg09333471**	-8.10E-03	1.16E-02	4.94E-01	1.84E-01	8.13E-02	3.54E-03	1.92E-01	4.19E-02	5.92E-03	HNSC
**cg00473134**	-1.12E-02	3.95E-03	5.94E-03*	6.47E-02	3.33E-02	1.45E-03	7.65E-02	1.67E-02	2.37E-03	HNSC
**cg03995857**	-7.61E-03	2.66E-03	6.11E-03*	7.85E-02	3.54E-02	1.54E-03	8.52E-02	1.64E-02	2.32E-03	HNSC
**cg07320646**	-2.07E-04	6.50E-04	7.52E-01	2.45E-02	4.07E-02	1.77E-03	2.11E-02	3.50E-03	4.95E-04	HNSC
**cg07293520**	-3.06E-03	1.37E-03	3.02E-02*	2.92E-02	3.91E-02	1.70E-03	2.91E-02	9.08E-03	1.28E-03	HNSC
**cg04770504**	3.14E-03	2.49E-03	2.13E-01	2.33E-02	3.60E-02	1.57E-03	1.74E-02	2.53E-03	3.58E-04	HNSC
**cg24805239**	-1.28E-03	6.01E-03	8.34E-01	6.53E-02	4.21E-02	1.83E-03	6.66E-02	1.73E-02	2.44E-03	HNSC
**cg01733570**	-1.51E-02	2.15E-02	4.91E-01	3.45E-01	1.46E-01	6.37E-03	3.60E-01	1.35E-01	1.90E-02	HNSC
**cg25723149**	4.25E-03	1.93E-02	8.29E-01	2.75E-01	1.32E-01	5.75E-03	2.71E-01	1.16E-01	1.64E-02	HNSC
**cg22804000**	5.29E-03	1.87E-02	7.81E-01	2.60E-01	1.27E-01	5.52E-03	2.54E-01	1.20E-01	1.69E-02	HNSC
**cg07504598**	1.33E-01	2.52E-02	3.49E-07*	4.79E-01	1.77E-01	7.68E-03	3.47E-01	1.30E-01	1.84E-02	HNSC
**cg15037663**	1.44E-01	2.64E-02	1.30E-07*	5.62E-01	1.83E-01	7.97E-03	4.18E-01	1.20E-01	1.70E-02	HNSC
**cg19706795**	1.63E-01	2.55E-02	6.45E-10*	6.66E-01	1.75E-01	7.62E-03	5.03E-01	1.39E-01	1.97E-02	HNSC
**cg20764575**	1.76E-01	2.74E-02	6.98E-10*	6.20E-01	1.89E-01	8.23E-03	4.45E-01	1.51E-01	2.13E-02	HNSC
**cg06301139**	-4.67E-02	2.02E-02	2.37E-02*	8.66E-01	1.60E-01	6.97E-03	9.20E-01	1.87E-02	2.65E-03	HNSC
**Probe**	**Mean Diff**	**Se Diff**	**KR_p-value**	**Mean TP**	**SD mean TP**	**SEM TP**	**Mean NT**	**SD mean NT**	**SEM NT**	**Tumour Type**

**cg17790129**	-8.43E-04	5.48E-03	8.78E-01	9.12E-01	6.85E-02	3.80E-03	9.13E-01	2.03E-02	1.61E-03	KIRC
**cg14205998**	-1.37E-03	1.55E-03	3.80E-01	9.30E-01	2.30E-02	1.28E-03	9.32E-01	2.11E-02	1.66E-03	KIRC
**cg04317854**	3.20E-02	1.20E-02	8.41E-03*	6.32E-01	1.65E-01	9.16E-03	6.09E-01	8.93E-02	7.06E-03	KIRC
**cg12922093**	-1.74E-02	4.40E-03	1.06E-04*	8.77E-01	5.62E-02	3.12E-03	8.95E-01	2.50E-02	1.98E-03	KIRC
**cg17569154**	-7.53E-02	9.36E-03	2.45E-14*	8.68E-01	1.19E-01	6.62E-03	9.43E-01	1.29E-02	1.02E-03	KIRC
**cg19260663**	-1.82E-02	2.53E-03	1.03E-11*	9.08E-01	3.60E-02	2.00E-03	9.31E-01	1.82E-02	1.44E-03	KIRC
**cg09333471**	-4.71E-03	5.44E-03	3.88E-01	1.68E-01	6.14E-02	3.41E-03	1.73E-01	4.83E-02	3.82E-03	KIRC
**cg00473134**	-2.67E-03	1.48E-03	7.37E-02	5.90E-02	1.70E-02	9.46E-04	6.15E-02	1.58E-02	1.25E-03	KIRC
**cg03995857**	1.74E-03	1.52E-03	2.55E-01	7.13E-02	1.79E-02	9.95E-04	6.82E-02	1.54E-02	1.22E-03	KIRC
**cg07320646**	4.10E-04	2.50E-04	1.03E-01	1.88E-02	5.69E-03	3.16E-04	1.59E-02	2.22E-03	1.76E-04	KIRC
**cg07293520**	7.20E-04	4.62E-04	1.22E-01	2.44E-02	5.77E-03	3.21E-04	2.29E-02	5.42E-03	4.28E-04	KIRC
**cg04770504**	8.80E-04	1.01E-03	3.85E-01	1.75E-02	1.25E-02	6.95E-04	1.66E-02	3.74E-03	2.96E-04	KIRC
**cg24805239**	-1.05E-02	2.57E-03	5.95E-05*	5.33E-02	2.80E-02	1.55E-03	6.39E-02	2.38E-02	1.88E-03	KIRC
**cg01733570**	-8.66E-02	1.06E-02	1.10E-14*	3.79E-01	1.31E-01	7.29E-03	4.66E-01	7.40E-02	5.85E-03	KIRC
**cg25723149**	1.66E-02	1.13E-02	1.43E-01	3.54E-01	1.44E-01	8.00E-03	3.38E-01	6.77E-02	5.35E-03	KIRC
**cg22804000**	-2.44E-02	1.06E-02	2.27E-02*	3.11E-01	1.31E-01	7.30E-03	3.36E-01	6.05E-02	4.78E-03	KIRC
**cg07504598**	8.74E-03	9.95E-03	3.81E-01	3.80E-01	1.30E-01	7.21E-03	3.75E-01	7.91E-02	6.25E-03	KIRC
**cg15037663**	-1.54E-02	9.58E-03	1.09E-01	4.48E-01	1.24E-01	6.86E-03	4.64E-01	6.40E-02	5.06E-03	KIRC
**cg19706795**	-1.71E-02	1.03E-02	9.92E-02	5.61E-01	1.35E-01	7.49E-03	5.80E-01	5.98E-02	4.73E-03	KIRC
**cg20764575**	2.81E-02	1.31E-02	3.31E-02*	6.45E-01	1.75E-01	9.72E-03	6.21E-01	6.73E-02	5.32E-03	KIRC
**cg06301139**	-1.24E-02	3.18E-03	1.30E-04*	9.32E-01	4.31E-02	2.39E-03	9.44E-01	1.24E-02	9.77E-04	KIRC
**cg26712096**	-1.20E-02	2.42E-03	1.37E-06*	9.30E-01	3.14E-02	1.74E-03	9.43E-01	1.66E-02	1.31E-03	KIRC
**Probe**	**Mean Diff**	**Se Diff**	**KR_p-value**	**Mean TP**	**SD mean TP**	**SEM TP**	**Mean NT**	**SD mean NT**	**SEM NT**	**Tumour Type**
**cg17790129**	8.64E-03	9.58E-03	3.75E-01	9.17E-01	7.89E-02	4.76E-03	9.08E-01	2.28E-02	3.40E-03	KIRP

**cg14205998**	-1.05E-03	1.85E-03	5.75E-01	9.30E-01	1.91E-02	1.15E-03	9.42E-01	1.09E-02	1.63E-03	KIRP
**cg04317854**	2.55E-01	2.12E-02	6.11E-24*	8.12E-01	1.43E-01	8.62E-03	5.58E-01	8.52E-02	1.27E-02	KIRP
**cg12922093**	1.79E-02	6.00E-03	4.00E-03*	8.98E-01	4.19E-02	2.53E-03	8.79E-01	2.83E-02	4.22E-03	KIRP
**cg17569154**	-1.38E-01	2.43E-02	3.35E-07*	7.68E-01	2.09E-01	1.26E-02	9.31E-01	1.81E-02	2.70E-03	KIRP
**cg19260663**	-2.29E-02	7.71E-03	3.96E-03*	9.04E-01	5.23E-02	3.15E-03	9.25E-01	2.82E-02	4.20E-03	KIRP
**cg09333471**	2.85E-03	1.74E-02	8.72E-01	1.75E-01	1.16E-01	6.97E-03	1.72E-01	3.54E-02	5.28E-03	KIRP
**cg00473134**	1.05E-02	1.57E-02	5.11E-01	7.65E-02	1.05E-01	6.32E-03	6.61E-02	1.17E-02	1.75E-03	KIRP
**cg03995857**	1.41E-02	1.64E-02	3.99E-01	9.02E-02	1.10E-01	6.65E-03	7.69E-02	1.24E-02	1.84E-03	KIRP
**cg07320646**	1.24E-02	1.83E-02	5.06E-01	4.25E-02	1.20E-01	7.24E-03	3.01E-02	6.35E-02	9.47E-03	KIRP
**cg07293520**	1.94E-02	1.54E-02	2.17E-01	4.26E-02	1.03E-01	6.23E-03	2.32E-02	4.53E-03	6.75E-04	KIRP
**cg04770504**	1.42E-02	1.19E-02	2.41E-01	3.12E-02	7.95E-02	4.79E-03	1.70E-02	3.11E-03	4.63E-04	KIRP
**cg24805239**	6.78E-03	1.46E-02	6.48E-01	6.90E-02	9.76E-02	5.89E-03	6.22E-02	1.69E-02	2.52E-03	KIRP
**cg01733570**	-4.55E-03	3.03E-02	8.83E-01	4.17E-01	2.02E-01	1.22E-02	4.22E-01	6.05E-02	9.02E-03	KIRP
**cg25723149**	4.42E-02	2.93E-02	1.39E-01	3.63E-01	1.95E-01	1.18E-02	3.19E-01	5.53E-02	8.24E-03	KIRP
**cg22804000**	3.40E-02	2.88E-02	2.47E-01	3.46E-01	1.92E-01	1.16E-02	3.12E-01	5.28E-02	7.88E-03	KIRP
**cg07504598**	5.31E-02	3.07E-02	9.06E-02	4.09E-01	2.10E-01	1.26E-02	3.67E-01	7.41E-02	1.11E-02	KIRP
**cg15037663**	1.26E-02	2.79E-02	6.58E-01	4.35E-01	1.93E-01	1.17E-02	4.36E-01	5.77E-02	8.60E-03	KIRP
**cg19706795**	6.11E-02	2.97E-02	4.43E-02*	6.08E-01	2.05E-01	1.24E-02	5.56E-01	5.35E-02	7.98E-03	KIRP
**cg20764575**	9.51E-02	2.80E-02	1.11E-03*	6.59E-01	2.09E-01	1.26E-02	5.84E-01	5.74E-02	8.56E-03	KIRP
**cg06301139**	-6.05E-03	3.30E-03	7.29E-02	9.30E-01	2.21E-02	1.33E-03	9.37E-01	1.67E-02	2.49E-03	KIRP
**cg26712096**	-8.25E-03	4.77E-03	9.15E-02	9.33E-01	4.02E-02	2.42E-03	9.49E-01	1.10E-02	1.64E-03	KIRP

**Probe**	**Mean Diff**	**Se Diff**	**KR_p-value**	**Mean TP**	**SD mean TP**	**SEM TP**	**Mean NT**	**SD mean NT**	**SEM NT**	**Tumour Type**
**cg17790129**	-2.05E-02	1.15E-02	7.99E-02	9.01E-01	8.23E-02	4.24E-03	9.23E-01	2.91E-02	4.12E-03	LIHC
**cg14205998**	-3.17E-02	8.87E-03	4.99E-04*	9.01E-01	6.24E-02	3.21E-03	9.33E-01	1.47E-02	2.08E-03	LIHC

**cg04317854**	-9.48E-02	2.48E-02	2.09E-04*	7.48E-01	1.78E-01	9.18E-03	8.43E-01	4.81E-02	6.80E-03	LIHC
**cg12922093**	-1.74E-01	2.78E-02	5.81E-09*	7.01E-01	2.10E-01	1.08E-02	8.64E-01	3.40E-02	4.80E-03	LIHC
**cg17569154**	-1.07E-01	2.43E-02	1.91E-05*	8.34E-01	1.72E-01	8.84E-03	9.41E-01	1.73E-02	2.44E-03	LIHC
**cg19260663**	-1.90E-01	3.09E-02	4.24E-09*	7.21E-01	2.19E-01	1.13E-02	9.10E-01	3.03E-02	4.29E-03	LIHC
**cg09333471**	-9.53E-03	2.82E-02	7.39E-01	3.79E-01	1.98E-01	1.02E-02	3.93E-01	9.63E-02	1.36E-02	LIHC
**cg00473134**	8.23E-02	2.25E-02	3.63E-04*	1.60E-01	1.60E-01	8.23E-03	8.05E-02	1.74E-02	2.47E-03	LIHC
**cg03995857**	8.43E-02	2.42E-02	6.60E-04*	1.85E-01	1.70E-01	8.76E-03	1.00E-01	4.03E-02	5.70E-03	LIHC
**cg07320646**	1.45E-02	1.30E-02	2.75E-01	3.88E-02	9.20E-02	4.74E-03	2.43E-02	9.55E-03	1.35E-03	LIHC
**cg07293520**	6.76E-03	6.59E-03	3.13E-01	3.10E-02	4.66E-02	2.40E-03	2.42E-02	4.17E-03	5.89E-04	LIHC
**cg04770504**	1.59E-02	1.06E-02	1.41E-01	3.50E-02	7.49E-02	3.86E-03	1.91E-02	2.56E-03	3.62E-04	LIHC
**cg24805239**	2.09E-02	1.35E-02	1.27E-01	7.46E-02	9.51E-02	4.90E-03	5.36E-02	1.46E-02	2.06E-03	LIHC
**cg01733570**	4.03E-02	3.31E-02	2.32E-01	3.54E-01	2.32E-01	1.19E-02	3.13E-01	8.70E-02	1.23E-02	LIHC
**cg25723149**	6.74E-02	3.28E-02	4.40E-02*	3.20E-01	2.30E-01	1.18E-02	2.53E-01	9.19E-02	1.30E-02	LIHC
**cg22804000**	7.39E-02	3.20E-02	2.35E-02*	3.03E-01	2.24E-01	1.15E-02	2.29E-01	8.22E-02	1.16E-02	LIHC
**cg07504598**	4.15E-02	3.40E-02	2.30E-01	3.85E-01	2.37E-01	1.22E-02	3.43E-01	9.71E-02	1.37E-02	LIHC
**cg15037663**	3.76E-02	3.48E-02	2.88E-01	4.80E-01	2.43E-01	1.25E-02	4.43E-01	1.12E-01	1.59E-02	LIHC
**cg19706795**	5.93E-02	3.30E-02	7.74E-02	5.66E-01	2.30E-01	1.18E-02	5.07E-01	1.09E-01	1.55E-02	LIHC
**cg20764575**	1.21E-01	3.61E-02	1.08E-03*	5.61E-01	2.49E-01	1.28E-02	4.40E-01	1.47E-01	2.08E-02	LIHC
**cg06301139**	-9.03E-02	2.38E-02	2.20E-04*	8.29E-01	1.68E-01	8.65E-03	9.20E-01	2.35E-02	3.32E-03	LIHC
**cg26712096**	-1.14E-01	2.92E-02	1.37E-04*	8.20E-01	2.07E-01	1.07E-02	9.35E-01	2.02E-02	2.86E-03	LIHC
**Probe**	**Mean Diff**	**Se Diff**	**KR_p-value**	**Mean TP**	**SD mean TP**	**SEM TP**	**Mean NT**	**SD mean NT**	**SEM NT**	**Tumour Type**
**cg17790129**	4.53E-02	1.46E-02	2.88E-03*	8.23E-01	1.03E-01	4.71E-03	7.72E-01	4.27E-02	7.55E-03	LUAD
**cg14205998**	-6.22E-03	8.15E-03	4.51E-01	8.59E-01	8.40E-02	3.86E-03	8.84E-01	2.99E-02	5.29E-03	LUAD
**cg04317854**	1.04E-01	1.70E-02	4.20E-08*	7.44E-01	1.17E-01	5.38E-03	6.26E-01	5.66E-02	1.00E-02	LUAD

**cg12922093**	-5.40E-02	1.70E-02	2.37E-03*	7.28E-01	1.32E-01	6.05E-03	8.04E-01	3.86E-02	6.82E-03	LUAD
**cg17569154**	-2.50E-02	1.33E-02	6.72E-02	8.21E-01	1.02E-01	4.68E-03	8.50E-01	3.57E-02	6.31E-03	LUAD
**cg19260663**	-5.61E-02	1.37E-02	1.37E-04*	7.84E-01	1.15E-01	5.28E-03	8.39E-01	4.04E-02	7.14E-03	LUAD
**cg09333471**	6.95E-02	1.93E-02	5.02E-04*	2.84E-01	1.15E-01	5.30E-03	2.18E-01	3.75E-02	6.63E-03	LUAD
**cg00473134**	4.78E-02	1.82E-02	1.01E-02*	1.48E-01	1.05E-01	4.84E-03	1.05E-01	2.65E-02	4.68E-03	LUAD
**cg03995857**	8.69E-02	2.28E-02	2.68E-04*	2.05E-01	1.46E-01	6.71E-03	1.35E-01	2.41E-02	4.25E-03	LUAD
**cg07320646**	6.97E-02	2.29E-02	3.49E-03*	1.05E-01	1.63E-01	7.52E-03	4.26E-02	1.51E-02	2.67E-03	LUAD
**cg07293520**	5.77E-02	1.96E-02	4.35E-03*	1.07E-01	1.25E-01	5.76E-03	5.70E-02	1.50E-02	2.65E-03	LUAD
**cg04770504**	7.31E-02	1.96E-02	4.28E-04*	1.20E-01	1.48E-01	6.82E-03	3.92E-02	1.20E-02	2.13E-03	LUAD
**cg24805239**	6.19E-02	1.93E-02	2.12E-03*	1.76E-01	1.36E-01	6.26E-03	1.18E-01	2.11E-02	3.73E-03	LUAD
**cg01733570**	-3.37E-02	2.09E-02	1.14E-01	4.00E-01	1.27E-01	5.86E-03	4.45E-01	5.60E-02	9.89E-03	LUAD
**cg25723149**	3.43E-02	2.14E-02	1.18E-01	3.66E-01	1.33E-01	6.09E-03	3.42E-01	5.47E-02	9.66E-03	LUAD
**cg22804000**	3.20E-02	2.01E-02	1.19E-01	3.42E-01	1.23E-01	5.66E-03	3.17E-01	5.48E-02	9.69E-03	LUAD
**cg07504598**	9.85E-02	1.65E-02	8.60E-08*	6.18E-01	1.17E-01	5.36E-03	5.06E-01	5.21E-02	9.21E-03	LUAD
**cg15037663**	8.46E-02	1.67E-02	2.67E-06*	6.77E-01	1.10E-01	5.06E-03	5.90E-01	4.82E-02	8.51E-03	LUAD
**cg19706795**	4.04E-02	1.59E-02	1.30E-02*	7.52E-01	8.95E-02	4.11E-03	7.11E-01	3.76E-02	6.65E-03	LUAD
**cg20764575**	8.99E-02	1.50E-02	8.18E-09*	7.40E-01	8.39E-02	3.86E-03	6.49E-01	5.86E-02	1.04E-02	LUAD
**cg06301139**	-1.24E-02	7.50E-03	1.04E-01	8.66E-01	7.25E-02	3.33E-03	8.82E-01	2.51E-02	4.44E-03	LUAD
**cg26712096**	-2.92E-02	1.36E-02	3.68E-02*	8.13E-01	1.20E-01	5.50E-03	8.59E-01	2.94E-02	5.20E-03	LUAD

**Probe**	**Mean Diff**	**Se Diff**	**KR_p-value**	**Mean TP**	**SD mean TP**	**SEM TP**	**Mean NT**	**SD mean NT**	**SEM NT**	**Tumour Type**
**cg17790129**	5.53E-02	1.33E-02	5.65E-05*	8.86E-01	8.48E-02	4.41E-03	8.31E-01	4.50E-02	6.94E-03	LUSC
**cg14205998**	-2.40E-02	6.55E-03	6.89E-04*	8.91E-01	8.96E-02	4.66E-03	9.36E-01	1.13E-02	1.74E-03	LUSC
**cg04317854**	1.17E-01	1.84E-02	1.12E-08*	7.76E-01	1.33E-01	6.92E-03	6.66E-01	7.54E-02	1.16E-02	LUSC
**cg12922093**	-3.20E-02	1.59E-02	4.96E-02*	7.95E-01	1.03E-01	5.33E-03	8.27E-01	3.16E-02	4.87E-03	LUSC

**cg17569154**	-6.77E-02	2.04E-02	1.28E-03*	8.36E-01	1.33E-01	6.90E-03	9.04E-01	2.98E-02	4.60E-03	LUSC
**cg19260663**	-3.45E-02	8.77E-03	2.92E-04*	8.63E-01	1.04E-01	5.38E-03	9.25E-01	1.71E-02	2.64E-03	LUSC
**cg09333471**	1.74E-02	1.27E-02	1.82E-01	1.92E-01	8.39E-02	4.36E-03	1.75E-01	3.68E-02	5.68E-03	LUSC
**cg00473134**	4.21E-03	6.25E-03	5.09E-01	6.70E-02	4.05E-02	2.11E-03	6.30E-02	1.32E-02	2.03E-03	LUSC
**cg03995857**	2.58E-03	7.32E-03	7.30E-01	8.02E-02	4.72E-02	2.46E-03	7.76E-02	1.22E-02	1.89E-03	LUSC
**cg07320646**	6.17E-03	6.33E-03	3.40E-01	2.44E-02	4.10E-02	2.13E-03	1.83E-02	2.75E-03	4.25E-04	LUSC
**cg07293520**	4.12E-03	5.70E-03	4.79E-01	2.80E-02	3.69E-02	1.92E-03	2.39E-02	5.69E-03	8.79E-04	LUSC
**cg04770504**	5.25E-03	5.04E-03	3.08E-01	2.27E-02	3.26E-02	1.69E-03	1.75E-02	3.11E-03	4.80E-04	LUSC
**cg24805239**	-8.05E-03	7.24E-03	2.76E-01	7.40E-02	4.67E-02	2.43E-03	8.21E-02	1.42E-02	2.19E-03	LUSC
**cg01733570**	1.35E-02	2.64E-02	6.16E-01	4.45E-01	1.71E-01	8.89E-03	4.36E-01	6.42E-02	9.91E-03	LUSC
**cg25723149**	5.21E-02	2.48E-02	4.01E-02*	3.54E-01	1.60E-01	8.32E-03	3.03E-01	5.28E-02	8.15E-03	LUSC
**cg22804000**	5.09E-02	2.35E-02	3.46E-02*	3.37E-01	1.51E-01	7.86E-03	2.86E-01	5.44E-02	8.39E-03	LUSC
**cg07504598**	3.95E-02	2.38E-02	1.05E-01	5.29E-01	1.69E-01	8.77E-03	5.04E-01	6.18E-02	9.54E-03	LUSC
**cg15037663**	4.89E-02	2.32E-02	4.03E-02*	6.21E-01	1.77E-01	9.20E-03	6.02E-01	5.07E-02	7.83E-03	LUSC
**cg19706795**	1.52E-02	1.82E-02	4.11E-01	7.17E-01	1.60E-01	8.33E-03	7.26E-01	4.99E-02	7.70E-03	LUSC
**cg20764575**	4.12E-02	2.17E-02	6.41E-02	6.70E-01	1.71E-01	8.87E-03	6.67E-01	5.46E-02	8.42E-03	LUSC
**cg06301139**	-2.60E-02	1.61E-02	1.15E-01	8.90E-01	1.14E-01	5.93E-03	9.20E-01	2.35E-02	3.63E-03	LUSC
**cg26712096**	-4.66E-02	1.40E-02	1.70E-03*	8.56E-01	1.39E-01	7.23E-03	9.25E-01	1.68E-02	2.59E-03	LUSC

Probe	Mean Diff	Se Diff	KR_p-value	Mean TP	SD mean TP	SEM TP	Mean NT	SD mean NT	SEM NT	Tumour Type
**cg17790129**	-7.74E-02	3.30E-02	3.93E-02*	7.87E-01	1.23E-01	9.10E-03	8.88E-01	2.69E-02	8.51E-03	PAAD
**cg14205998**	-1.09E-02	6.51E-03	1.30E-01	9.07E-01	3.23E-02	2.38E-03	9.32E-01	1.18E-02	3.73E-03	PAAD
**cg04317854**	6.29E-02	3.31E-02	8.46E-02	7.69E-01	1.03E-01	7.56E-03	7.06E-01	8.95E-02	2.83E-02	PAAD
**cg12922093**	1.23E-02	1.91E-02	5.39E-01	8.02E-01	1.13E-01	8.30E-03	8.27E-01	6.29E-02	1.99E-02	PAAD
**cg17569154**	-9.33E-02	2.89E-02	8.15E-03*	7.67E-01	1.30E-01	9.62E-03	8.80E-01	4.91E-02	1.55E-02	PAAD

**cg19260663**	6.54E-03	8.05E-03	4.38E-01	8.44E-01	1.10E-01	8.08E-03	9.00E-01	2.76E-02	8.73E-03	PAAD
**cg09333471**	-3.88E-02	3.09E-02	2.49E-01	3.53E-01	1.28E-01	9.41E-03	3.78E-01	7.50E-02	2.37E-02	PAAD
**cg00473134**	5.66E-02	2.95E-02	8.61E-02	1.54E-01	1.33E-01	9.84E-03	8.73E-02	7.56E-02	2.39E-02	PAAD
**cg03995857**	7.30E-02	3.42E-02	6.00E-02	1.85E-01	1.60E-01	1.18E-02	1.10E-01	1.14E-01	3.60E-02	PAAD
**cg07320646**	3.57E-05	7.24E-04	9.62E-01	4.93E-02	1.05E-01	7.77E-03	1.73E-02	3.78E-03	1.20E-03	PAAD
**cg07293520**	1.18E-02	1.04E-02	2.89E-01	4.46E-02	7.08E-02	5.22E-03	2.14E-02	5.73E-03	1.81E-03	PAAD
**cg04770504**	3.86E-02	2.46E-02	1.54E-01	7.53E-02	1.15E-01	8.47E-03	2.83E-02	3.39E-02	1.07E-02	PAAD
**cg24805239**	3.78E-02	2.72E-02	2.02E-01	1.25E-01	1.21E-01	8.94E-03	6.61E-02	4.58E-02	1.45E-02	PAAD
**cg01733570**	-2.13E-01	3.84E-02	1.17E-04*	5.18E-01	1.69E-01	1.24E-02	6.97E-01	7.59E-02	2.40E-02	PAAD
**cg25723149**	-1.30E-01	3.07E-02	1.55E-03*	4.87E-01	1.65E-01	1.21E-02	5.66E-01	4.96E-02	1.57E-02	PAAD
**cg22804000**	-1.25E-01	3.36E-02	3.40E-03*	4.67E-01	1.59E-01	1.17E-02	5.63E-01	7.97E-02	2.52E-02	PAAD
**cg07504598**	1.93E-02	2.96E-02	5.40E-01	6.05E-01	1.30E-01	9.58E-03	5.79E-01	6.95E-02	2.20E-02	PAAD
**cg15037663**	-3.38E-02	2.53E-02	2.19E-01	6.70E-01	1.16E-01	8.52E-03	7.01E-01	4.35E-02	1.37E-02	PAAD
**cg19706795**	-4.78E-02	1.84E-02	2.65E-02*	7.71E-01	8.91E-02	6.57E-03	8.15E-01	5.85E-02	1.85E-02	PAAD
**cg20764575**	-4.35E-02	2.88E-02	1.71E-01	7.52E-01	9.15E-02	6.74E-03	8.00E-01	7.10E-02	2.25E-02	PAAD
**cg06301139**	3.96E-03	5.34E-03	4.81E-01	9.29E-01	3.82E-02	2.82E-03	9.43E-01	1.64E-02	5.19E-03	PAAD
**cg26712096**	-4.40E-03	9.24E-03	6.48E-01	8.94E-01	6.34E-02	4.67E-03	9.30E-01	1.34E-02	4.22E-03	PAAD

Probe	Mean Diff	Se Diff	KR_p-value	Mean TP	SD mean TP	SEM TP	Mean NT	SD mean NT	SEM NT	Tumour Type
**cg17790129**	-3.15E-03	3.81E-03	4.16E-01	9.34E-01	2.60E-02	1.16E-03	9.38E-01	2.21E-02	3.12E-03	PRAD
**cg14205998**	-2.84E-03	2.59E-03	2.78E-01	9.26E-01	2.62E-02	1.17E-03	9.41E-01	1.18E-02	1.68E-03	PRAD
**cg04317854**	3.95E-02	1.50E-02	9.88E-03*	7.07E-01	1.07E-01	4.76E-03	6.68E-01	8.95E-02	1.27E-02	PRAD
**cg12922093**	-7.26E-02	1.43E-02	1.20E-06*	8.33E-01	1.05E-01	4.71E-03	9.06E-01	2.70E-02	3.82E-03	PRAD
**cg17569154**	1.17E-02	1.03E-02	2.67E-01	8.54E-01	8.10E-02	3.62E-03	8.41E-01	7.32E-02	1.04E-02	PRAD
**cg19260663**	-9.24E-02	1.82E-02	1.04E-06*	8.25E-01	1.31E-01	5.86E-03	9.19E-01	3.87E-02	5.48E-03	PRAD

**cg09333471**	2.46E-01	1.98E-02	3.11E-30*	4.97E-01	1.37E-01	6.11E-03	2.51E-01	8.65E-02	1.22E-02	PRAD
**cg00473134**	1.50E-01	1.66E-02	4.32E-14*	2.45E-01	1.48E-01	6.59E-03	8.30E-02	4.73E-02	6.68E-03	PRAD
**cg03995857**	1.06E-01	1.44E-02	3.04E-10*	2.21E-01	1.54E-01	6.89E-03	8.87E-02	4.92E-02	6.96E-03	PRAD
**cg07320646**	-2.53E-04	1.40E-03	8.57E-01	2.68E-02	4.47E-02	2.00E-03	2.01E-02	3.32E-03	4.69E-04	PRAD
**cg07293520**	5.93E-04	9.60E-04	5.39E-01	3.33E-02	4.63E-02	2.07E-03	2.64E-02	5.64E-03	7.97E-04	PRAD
**cg04770504**	7.17E-02	1.51E-02	8.94E-06*	1.05E-01	1.32E-01	5.90E-03	1.97E-02	6.60E-03	9.33E-04	PRAD
**cg24805239**	1.51E-01	1.77E-02	2.81E-13*	2.60E-01	1.52E-01	6.78E-03	1.00E-01	4.45E-02	6.29E-03	PRAD
**cg01733570**	1.18E-01	1.09E-02	7.86E-20*	7.93E-01	8.24E-02	3.68E-03	6.74E-01	8.17E-02	1.16E-02	PRAD
**cg25723149**	1.54E-01	1.32E-02	9.38E-21*	7.58E-01	1.07E-01	4.76E-03	6.00E-01	8.93E-02	1.26E-02	PRAD
**cg22804000**	1.55E-01	1.39E-02	5.61E-20*	7.42E-01	1.10E-01	4.90E-03	5.89E-01	8.33E-02	1.18E-02	PRAD
**cg07504598**	1.21E-01	1.39E-02	2.16E-15*	7.24E-01	1.01E-01	4.51E-03	6.03E-01	7.84E-02	1.11E-02	PRAD
**cg15037663**	7.42E-02	1.11E-02	2.00E-10*	7.92E-01	7.71E-02	3.44E-03	7.18E-01	7.19E-02	1.02E-02	PRAD
**cg19706795**	4.06E-02	7.75E-03	5.78E-07*	8.37E-01	5.50E-02	2.46E-03	7.97E-01	5.69E-02	8.04E-03	PRAD
**cg20764575**	2.98E-02	8.51E-03	6.22E-04*	8.26E-01	5.75E-02	2.57E-03	7.97E-01	5.66E-02	8.00E-03	PRAD
**cg06301139**	-9.23E-03	2.36E-03	1.30E-04*	9.37E-01	1.62E-02	7.21E-04	9.46E-01	1.26E-02	1.79E-03	PRAD
**cg26712096**	-7.43E-02	1.42E-02	8.08E-07*	8.29E-01	1.12E-01	4.98E-03	9.08E-01	4.26E-02	6.02E-03	PRAD
**Probe**	**Mean Diff**	**Se Diff**	**KR_p-value**	**Mean TP**	**SD mean TP**	**SEM TP**	**Mean NT**	**SD mean NT**	**SEM NT**	**Tumour Type**
**cg17790129**	6.87E-03	3.82E-03	7.71E-02	9.32E-01	4.57E-02	2.03E-03	9.23E-01	5.62E-02	7.51E-03	THCA
**cg14205998**	3.58E-04	2.82E-03	9.00E-01	9.33E-01	2.09E-02	9.29E-04	9.33E-01	1.55E-02	2.07E-03	THCA
**cg04317854**	7.74E-02	1.58E-02	3.47E-06*	8.10E-01	1.32E-01	5.88E-03	7.33E-01	8.96E-02	1.20E-02	THCA
**cg12922093**	7.66E-03	4.19E-03	7.28E-02	9.14E-01	4.43E-02	1.97E-03	9.08E-01	3.38E-02	4.51E-03	THCA
**cg17569154**	-1.17E-03	1.94E-03	5.50E-01	9.45E-01	1.63E-02	7.24E-04	9.46E-01	1.27E-02	1.70E-03	THCA
**cg19260663**	4.37E-03	4.88E-03	3.77E-01	9.22E-01	3.57E-02	1.59E-03	9.17E-01	2.25E-02	3.01E-03	THCA
**cg09333471**	3.06E-03	1.19E-02	7.99E-01	1.90E-01	9.60E-02	4.27E-03	1.89E-01	4.02E-02	5.38E-03	THCA

**cg00473134**	-2.36E-04	2.17E-03	9.14E-01	5.97E-02	2.57E-02	1.14E-03	6.11E-02	1.70E-02	2.27E-03	THCA
**cg03995857**	-2.68E-03	2.07E-03	2.01E-01	7.56E-02	2.15E-02	9.57E-04	8.03E-02	1.72E-02	2.29E-03	THCA
**cg07320646**	6.10E-04	8.68E-04	4.88E-01	2.20E-02	7.09E-03	3.15E-04	2.28E-02	7.02E-03	9.38E-04	THCA
**cg07293520**	-7.57E-04	7.49E-04	3.19E-01	2.52E-02	6.33E-03	2.81E-04	2.70E-02	1.04E-02	1.39E-03	THCA
**cg04770504**	-8.18E-04	4.94E-04	1.04E-01	2.01E-02	1.21E-02	5.38E-04	2.06E-02	4.42E-03	5.90E-04	THCA
**cg24805239**	2.82E-03	6.64E-03	6.75E-01	8.67E-02	5.68E-02	2.52E-03	8.33E-02	1.74E-02	2.32E-03	THCA
**cg01733570**	-2.16E-01	2.19E-02	1.86E-17*	3.47E-01	1.81E-01	8.06E-03	5.63E-01	6.50E-02	8.69E-03	THCA
**cg25723149**	-5.97E-02	2.12E-02	6.06E-03*	3.97E-01	1.75E-01	7.80E-03	4.56E-01	6.75E-02	9.02E-03	THCA
**cg22804000**	-8.32E-02	2.32E-02	4.77E-04*	4.01E-01	1.80E-01	7.98E-03	4.88E-01	4.87E-02	6.50E-03	THCA
**cg07504598**	6.78E-02	2.48E-02	7.45E-03*	5.65E-01	1.88E-01	8.36E-03	5.03E-01	9.60E-02	1.28E-02	THCA
**cg15037663**	3.09E-02	2.49E-02	2.22E-01	6.82E-01	1.85E-01	8.21E-03	6.51E-01	7.46E-02	9.96E-03	THCA
**cg19706795**	2.59E-02	1.30E-02	5.14E-02	8.35E-01	9.95E-02	4.43E-03	8.12E-01	4.31E-02	5.76E-03	THCA
**cg20764575**	4.70E-02	1.35E-02	6.29E-04*	8.48E-01	1.00E-01	4.44E-03	8.01E-01	4.77E-02	6.37E-03	THCA
**cg06301139**	2.24E-03	1.56E-03	1.59E-01	9.48E-01	1.13E-02	5.02E-04	9.46E-01	9.10E-03	1.22E-03	THCA
**cg26712096**	-2.22E-04	4.01E-03	9.57E-01	9.39E-01	2.96E-02	1.32E-03	9.39E-01	1.65E-02	2.21E-03	THCA
**Probe**	**Mean Diff**	**Se Diff**	**KR_p-value**	**Mean TP**	**SD mean TP**	**SEM TP**	**Mean NT**	**SD mean NT**	**SEM NT**	**Tumour Type**
**cg17790129**	-4.11E-02	1.72E-02	2.04E-02*	8.49E-01	1.41E-01	6.76E-03	8.96E-01	7.41E-02	1.09E-02	UCEC
**cg14205998**	-1.41E-02	6.63E-03	3.71E-02*	9.17E-01	4.63E-02	2.21E-03	9.33E-01	3.10E-02	4.57E-03	UCEC
**cg04317854**	2.79E-02	2.44E-02	2.61E-01	7.63E-01	1.75E-01	8.37E-03	7.45E-01	7.15E-02	1.05E-02	UCEC
**cg12922093**	-3.80E-02	1.46E-02	1.13E-02*	8.53E-01	1.02E-01	4.87E-03	8.92E-01	4.08E-02	6.01E-03	UCEC
**cg17569154**	1.43E-01	1.52E-02	8.46E-19*	8.94E-01	8.92E-02	4.26E-03	7.51E-01	1.60E-01	2.36E-02	UCEC
**cg19260663**	-6.34E-02	1.76E-02	4.21E-04*	8.42E-01	1.18E-01	5.65E-03	9.06E-01	4.34E-02	6.40E-03	UCEC
**cg09333471**	9.62E-02	2.39E-02	1.07E-04*	3.32E-01	1.72E-01	8.23E-03	2.37E-01	7.82E-02	1.15E-02	UCEC
**cg00473134**	3.23E-02	1.41E-02	2.70E-02*	1.27E-01	1.42E-01	6.77E-03	8.19E-02	2.47E-02	3.64E-03	UCEC

**cg03995857**	1.82E-02	1.01E-02	7.78E-02	1.44E-01	1.71E-01	8.15E-03	9.17E-02	2.23E-02	3.29E-03	UCEC
**cg07320646**	-6.43E-05	2.71E-03	9.81E-01	6.51E-02	1.55E-01	7.41E-03	2.84E-02	1.04E-02	1.54E-03	UCEC
**cg07293520**	-7.86E-04	3.46E-03	8.22E-01	6.05E-02	1.34E-01	6.40E-03	2.81E-02	6.46E-03	9.52E-04	UCEC
**cg04770504**	1.99E-02	8.87E-03	3.04E-02*	7.00E-02	1.41E-01	6.73E-03	2.01E-02	3.06E-03	4.51E-04	UCEC
**cg24805239**	2.91E-02	1.59E-02	7.40E-02	1.18E-01	1.55E-01	7.42E-03	7.00E-02	2.61E-02	3.86E-03	UCEC
**cg01733570**	-7.69E-02	3.23E-02	1.98E-02*	4.95E-01	2.25E-01	1.08E-02	5.69E-01	1.06E-01	1.56E-02	UCEC
**cg25723149**	-6.37E-02	3.15E-02	4.80E-02*	4.01E-01	2.26E-01	1.08E-02	4.61E-01	8.97E-02	1.32E-02	UCEC
**cg22804000**	-8.24E-02	2.94E-02	6.50E-03*	3.95E-01	2.15E-01	1.03E-02	4.73E-01	8.66E-02	1.28E-02	UCEC
**cg07504598**	-8.50E-02	2.88E-02	4.16E-03*	3.94E-01	2.05E-01	9.82E-03	4.76E-01	9.38E-02	1.38E-02	UCEC
**cg15037663**	-1.23E-01	2.84E-02	3.42E-05*	4.45E-01	2.06E-01	9.83E-03	5.61E-01	9.34E-02	1.38E-02	UCEC
**cg19706795**	-8.73E-02	2.75E-02	2.13E-03*	5.65E-01	2.02E-01	9.65E-03	6.55E-01	8.46E-02	1.25E-02	UCEC
**cg20764575**	-1.42E-01	3.01E-02	8.79E-06*	5.47E-01	2.31E-01	1.10E-02	6.95E-01	8.30E-02	1.22E-02	UCEC
**cg06301139**	-5.89E-02	1.39E-02	5.83E-05*	8.80E-01	1.07E-01	5.13E-03	9.45E-01	9.68E-03	1.43E-03	UCEC
**cg26712096**	-9.27E-02	2.27E-02	6.61E-05*	8.41E-01	1.54E-01	7.34E-03	9.34E-01	1.86E-02	2.74E-03	UCEC

TP = primary tumour, NT = normal tissue, Mean diff = difference in means (mean NT − mean TP), SE = standard error of difference of means, SD = standard deviation, SEM = standard error of the mean. * represents significant results for which p-value < 0.05. Light shaded rows represent intragenic CpGs while dark shaded rows represent putative promoter CpGs.

Table S2. Table of the individual dataset analysis AUC values that satisfy the specified thresholds (minimum average AUC = 0.84 and minimum AUC threshold = 0.80). NA values are tumour types for which the probe combination did not meet the AUC thresholds.

Probe Combination	BLCA	BRCA	CRC	ESCA	HNSC	KIRC	KIRP	LIHC	LUAD	LUSC	PAAD	PRAD	THCA	UCEC
**cg00473134+cg04317854+cg17569154**	NA	0.86	0.86	NA	0.96	0.80	0.94	0.89	0.89	0.90	NA	0.90	NA	NA
**cg00473134+cg04317854+cg19260663**	0.90	0.91	0.91	0.83	0.82	NA	0.91	0.89	0.92	0.92	NA	0.93	NA	NA
**cg00473134+cg04317854+cg26712096**	0.84	0.91	0.88	NA	0.89	NA	0.93	0.83	0.92	0.92	0.81	0.93	NA	0.81
**cg01733570+cg04317854+cg06301139**	NA	0.83	0.85	NA	0.85	NA	0.91	NA	0.89	0.87	0.84	0.89	0.88	0.84
**cg01733570+cg04317854+cg12922093**	NA	0.88	0.86	0.82	0.87	0.81	0.92	NA	0.92	0.86	0.82	0.90	0.88	NA

**cg01733570+cg04317854+cg14205998**	0.86	0.85	0.88	0.81	0.84	NA	0.92	NA	0.88	0.93	0.89	0.86	0.87	NA
**cg01733570+cg04317854+cg17569154**	NA	0.86	NA	0.81	0.96	0.87	0.93	0.86	0.89	0.90	0.83	0.84	0.88	0.81
**cg01733570+cg04317854+cg19260663**	0.90	0.91	0.84	0.89	NA	0.81	0.91	0.86	0.92	0.92	0.90	0.89	0.87	NA
**cg01733570+cg04317854+cg26712096**	0.85	0.90	NA	NA	0.89	NA	0.93	NA	0.92	0.92	0.87	0.88	0.88	0.82
**cg01733570+cg04770504+cg17569154**	NA	0.87	0.96	NA	0.92	0.86	0.81	0.86	0.81	NA	0.88	0.89	0.86	0.88
**cg01733570+cg17569154+cg19260663**	0.89	0.91	0.83	NA	0.92	0.89	0.86	0.91	NA	0.84	0.93	0.88	0.86	0.93
**cg03995857+cg04317854+cg17569154**	NA	0.85	0.90	0.82	0.96	NA	0.93	0.88	0.90	0.90	NA	0.85	NA	NA
**cg03995857+cg04317854+cg19260663**	0.89	0.90	0.92	0.86	NA	NA	0.91	0.88	0.93	0.92	NA	0.90	NA	NA
**cg04317854+cg04770504+cg17569154**	NA	0.84	0.92	0.84	0.96	0.80	0.94	0.85	0.93	0.89	NA	0.84	NA	0.81
**cg04317854+cg04770504+cg19260663**	0.91	0.90	0.94	NA	0.81	NA	0.91	0.87	0.92	0.92	NA	0.87	NA	NA
**cg04317854+cg04770504+cg26712096**	0.86	0.89	0.92	0.84	0.91	NA	0.93	0.80	0.92	0.92	NA	0.90	NA	0.83
**cg04317854+cg07293520+cg19260663**	0.89	0.88	0.92	0.85	NA	NA	0.92	0.87	0.92	0.92	0.83	0.80	NA	NA
**cg04317854+cg07320646+cg19260663**	0.90	0.88	0.92	0.86	NA	NA	0.92	0.87	0.91	0.93	0.86	0.82	NA	NA
**cg04317854+cg07320646+cg26712096**	0.86	0.86	0.90	0.81	0.89	NA	0.93	NA	0.92	0.93	0.84	0.82	NA	0.82
**cg04317854+cg07504598+cg19260663**	0.92	0.93	0.88	0.83	0.84	NA	0.92	0.87	0.93	0.93	NA	0.87	NA	NA
**cg04317854+cg07504598+cg26712096**	0.91	0.94	0.83	NA	0.92	NA	0.94	0.82	0.94	0.91	0.82	0.87	NA	0.80
**cg04317854+cg09333471+cg17569154**	NA	0.91	0.84	NA	0.96	0.80	0.94	0.85	0.91	0.90	0.81	0.93	NA	NA
**cg04317854+cg09333471+cg19260663**	0.91	0.93	0.89	0.88	NA	NA	0.93	0.86	0.92	0.93	NA	0.93	NA	NA
**cg04317854+cg09333471+cg26712096**	0.83	0.94	0.84	NA	0.90	NA	0.93	NA	0.92	0.91	0.80	0.93	NA	0.84
**cg04317854+cg12922093+cg17569154**	0.83	0.83	NA	0.81	0.96	0.80	0.95	0.85	0.93	0.91	NA	0.81	NA	0.83
**cg04317854+cg12922093+cg19260663**	0.90	0.87	0.83	0.86	0.88	NA	0.90	0.85	0.92	0.93	NA	0.85	NA	NA
**cg04317854+cg14205998+cg17569154**	0.89	NA	0.80	NA	0.96	NA	0.95	0.88	0.90	0.94	0.87	NA	NA	0.82
**cg04317854+cg14205998+cg19260663**	0.90	0.86	0.84	0.84	0.85	NA	0.92	0.86	0.92	0.95	0.82	0.84	NA	NA
**cg04317854+cg15037663+cg19260663**	0.91	0.92	0.83	0.86	0.84	NA	0.92	0.88	0.94	0.92	NA	0.85	NA	NA
**cg04317854+cg17569154+cg19260663**	0.90	0.86	NA	0.85	0.96	0.86	0.95	0.89	0.91	0.94	0.84	0.82	NA	0.90

**cg04317854+cg17569154+cg24805239**	NA	0.87	0.90	NA	0.96	0.83	0.94	0.85	0.91	0.90	0.82	0.87	NA	NA
**cg04317854+cg17569154+cg26712096**	0.86	0.86	NA	NA	0.96	0.82	0.95	0.87	0.92	0.92	0.84	0.83	NA	0.92
**cg04317854+cg19260663+cg19706795**	0.90	0.93	0.86	0.82	0.86	NA	0.91	0.88	0.92	0.93	0.82	0.84	NA	NA
**cg04317854+cg19260663+cg20764575**	0.92	0.93	NA	0.86	0.86	NA	0.92	0.90	0.94	0.92	NA	0.82	NA	NA
**cg04317854+cg19260663+cg24805239**	0.91	0.91	0.93	0.82	0.81	NA	0.91	0.87	0.91	0.92	NA	0.91	NA	NA
**cg04317854+cg19260663+cg25723149**	0.90	0.92	0.84	0.84	NA	NA	0.92	0.87	0.90	0.93	0.83	0.89	NA	NA
**cg04317854+cg19260663+cg26712096**	0.92	0.89	NA	NA	0.90	NA	0.93	0.86	0.92	0.94	0.83	0.85	NA	NA
**cg04317854+cg24805239+cg26712096**	0.88	0.91	0.89	NA	0.89	NA	0.93	0.81	0.92	0.91	0.81	0.92	NA	0.81
**cg17569154+cg19260663+cg20764575**	0.90	0.93	NA	NA	0.94	0.87	0.89	0.92	0.88	0.85	0.80	0.81	NA	0.92

Table S3. Summary of the RNA-seq expression levels in the different sample types and across the different tumour types.

Sample Type	Tumour Type	N	Mean	SD
NT	BLCA	19	8.506	0.982
NT	BRCA	113	8.206	0.448
NT	CRC	51	5.885	1.213
NT	ESCA	11	7.226	1.220
NT	HNSC	44	7.516	0.830
NT	KIRC	72	8.914	0.405
NT	KIRP	32	8.781	0.510
NT	LIHC	50	7.122	0.558
NT	LUAD	59	7.507	0.731
NT	LUSC	51	7.623	0.544
NT	PAAD	4	8.740	1.095
NT	PRAD	52	7.636	0.715
NT	THCA	59	9.004	0.379
NT	UCEC	24	9.132	0.766
TP	BLCA	408	7.709	1.854
TP	BRCA	1095	7.156	0.852
TP	CRC	379	5.515	1.494
TP	ESCA	184	7.419	2.023
TP	HNSC	520	9.422	1.275
TP	KIRC	533	8.753	0.945
TP	KIRP	290	9.376	1.169
TP	LIHC	371	7.121	1.799
TP	LUAD	515	7.676	1.315
TP	LUSC	502	8.536	1.302
TP	PAAD	178	7.963	1.037
TP	PRAD	497	6.718	0.868
TP	THCA	505	8.873	0.674
TP	UCEC	176	7.021	1.211

TP = primary tumour, NT = normal tissue, N = sample size, SD= standard deviation.

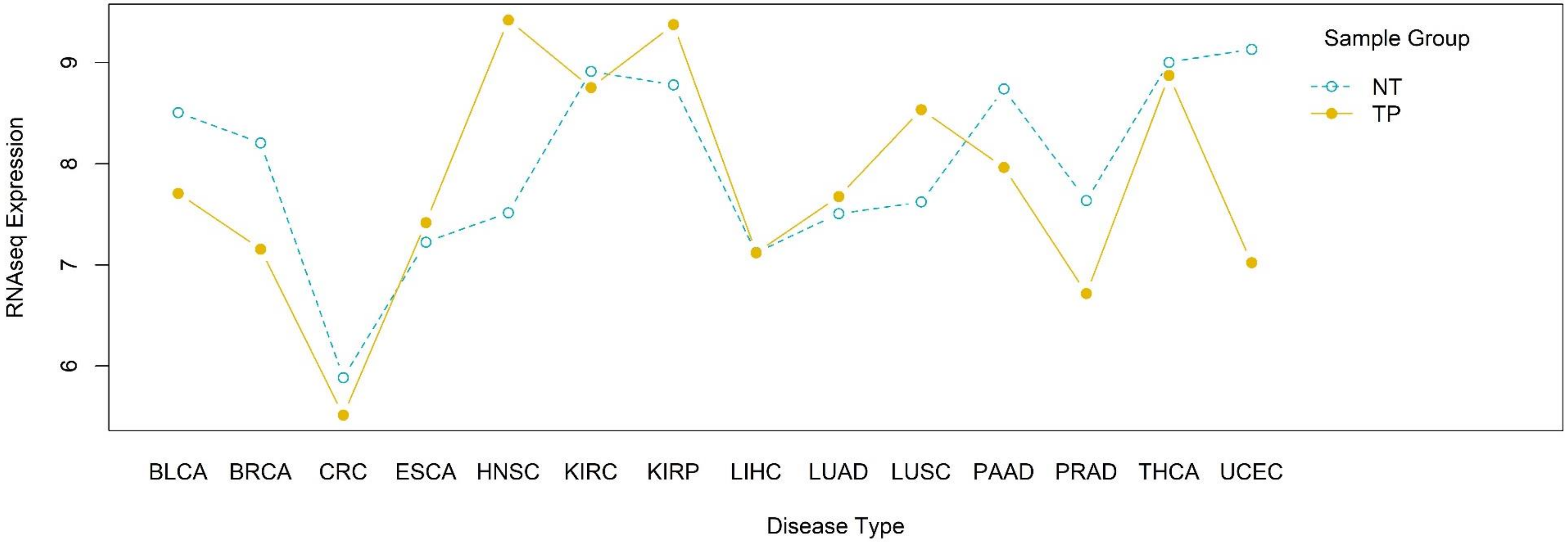

Figure S1. Interaction plot for the RNA-seq expression data showing the differences in expression levels between tumour and normal samples across the different tumour types.

Table S4. Table of the linear regression results for the analysis of RNA-seq expression and methylation.

probes	BLCA_slope	BLCA_*p*-value	BRCA_slope	BRCA_*p*-value	CRC_slope	CRC_*p*-value	ESCA_slope	ESCA_*p*-value	HNSC_slope	HNSC_*p*-value	KIRC_slope	KIRC_*p*-value	KIRP_slope	KIRP_*p*-value
cg00473134	0.087	0.848	0.005	0.986	0.044	0.929	-0.182	0.813	-1.527	0.038	0.146	0.783	-0.205	0.769
cg01733570	1.498	0.021	-0.063	0.850	0.304	0.523	2.181	0.101	3.180	0.037	0.945	0.583	6.252	0.033
cg03995857	2.121	0.000	0.373	0.182	-1.684	0.011	0.548	0.558	0.643	0.367	0.738	0.003	1.538	0.000
cg04317854	1.027	0.131	-0.133	0.650	-0.366	0.422	1.493	0.175	2.541	0.001	-0.356	0.620	-3.556	0.010
cg04770504	-0.533	0.155	0.048	0.828	0.385	0.358	-0.492	0.388	-1.578	0.000	-1.823	0.000	-0.802	0.003

cg06301139	0.932	0.106	0.794	0.009	0.646	0.220	0.046	0.970	1.274	0.262	0.163	0.877	-0.445	0.630
cg07293520	-1.864	0.030	-0.365	0.145	-0.703	0.269	-3.696	0.021	0.048	0.960	0.293	0.699	-3.137	0.002
cg07320646	-2.580	0.020	0.183	0.519	-0.679	0.238	1.695	0.313	-6.696	0.004	-1.751	0.406	5.594	0.019
cg07504598	-0.206	0.846	-0.108	0.679	-0.385	0.449	-1.136	0.674	6.051	0.013	-4.141	0.065	-6.629	0.012
cg09333471	0.995	0.287	0.245	0.377	-0.569	0.285	-2.871	0.331	-4.243	0.048	7.867	0.292	1.514	0.396
cg12922093	0.075	0.949	-0.113	0.753	0.781	0.184	0.986	0.631	1.286	0.413	-9.407	0.114	-4.466	0.468
cg14205998	-0.067	0.951	0.563	0.114	1.424	0.076	5.632	0.132	-0.150	0.955	-3.402	0.370	9.273	0.250
cg15037663	0.954	0.395	-0.353	0.307	-0.350	0.667	-1.620	0.511	-0.221	0.914	2.028	0.208	-6.609	0.022
cg17569154	-1.132	0.088	0.371	0.162	0.485	0.468	2.400	0.057	0.312	0.753	-0.273	0.538	0.664	0.276
cg17790129	-1.823	0.095	-0.951	0.025	-0.962	0.287	-0.902	0.673	-1.314	0.366	-2.581	0.000	1.891	0.049
cg19260663	1.146	0.316	0.130	0.736	-0.397	0.616	-6.030	0.005	-2.180	0.161	2.176	0.003	-1.942	0.063
cg19706795	-1.087	0.072	-0.254	0.399	0.633	0.202	-0.113	0.914	0.338	0.544	0.511	0.259	0.245	0.601
cg20764575	0.436	0.596	0.489	0.112	-0.354	0.568	2.244	0.133	0.492	0.528	0.633	0.360	0.855	0.219
cg22804000	-0.205	0.870	-0.093	0.830	0.334	0.684	-0.316	0.870	0.600	0.535	-0.152	0.830	-1.327	0.139
cg24805239	0.403	0.637	-0.362	0.258	-2.086	0.021	0.937	0.523	0.124	0.875	-0.313	0.337	-0.459	0.404
cg25723149	1.277	0.146	-0.085	0.792	0.746	0.180	-2.661	0.027	-2.412	0.000	-2.560	0.012	-4.202	0.076
cg26712096	1.136	0.034	0.667	0.019	2.781	0.000	1.357	0.120	3.547	0.000	3.766	0.007	1.169	0.454

probes	LIHC_slope	LIHC_*p*-value	LUAD_slope	LUAD_*p*-value	LUSC_slope	LUSC_*p*-value	PAAD_slope	PAAD_*p*-value	PRAD_slope	PRAD_*p*-value	THCA_slope	THCA_*p*-value	UCEC_slope	UCEC_*p*-value
cg00473134	-2.917	0.000	0.459	0.512	-1.923	0.024	0.005	0.992	-0.170	0.896	-0.017	0.972	1.299	0.054
cg01733570	0.782	0.468	-0.590	0.528	0.322	0.754	4.814	0.016	-0.083	0.952	-1.810	0.149	-2.340	0.367
cg03995857	1.453	0.005	1.462	0.006	2.465	0.000	0.679	0.280	0.601	0.090	0.408	0.030	-0.005	0.994
cg04317854	0.740	0.204	0.063	0.920	1.727	0.035	0.791	0.174	-0.818	0.081	-0.645	0.283	0.663	0.618
cg04770504	-0.514	0.461	-0.639	0.298	-1.173	0.039	1.048	0.064	-0.329	0.495	-3.435	0.053	-2.043	0.021
cg06301139	-0.699	0.214	2.124	0.004	1.610	0.075	1.603	0.013	0.652	0.091	1.764	0.059	2.265	0.017
cg07293520	-1.598	0.000	-1.525	0.068	-2.353	0.016	-1.803	0.017	-0.293	0.441	0.117	0.697	-2.561	0.002
cg07320646	0.024	0.969	-0.002	0.999	0.988	0.702	-0.184	0.830	-0.020	0.954	-2.153	0.137	0.714	0.691
cg07504598	-0.826	0.130	-0.944	0.286	-2.938	0.206	1.149	0.126	-0.112	0.726	1.390	0.367	-2.677	0.102
cg09333471	0.148	0.904	0.759	0.366	-9.670	0.173	4.161	0.001	-1.840	0.212	0.251	0.952	1.412	0.599
cg12922093	4.882	0.022	0.220	0.845	9.063	0.279	-6.404	0.001	-0.154	0.918	-1.629	0.669	0.280	0.926
cg14205998	-1.054	0.522	-1.217	0.140	2.376	0.539	1.186	0.265	0.462	0.305	-3.949	0.185	-4.399	0.035
cg15037663	0.317	0.792	0.512	0.617	1.523	0.545	-2.541	0.020	0.531	0.239	-1.191	0.025	4.664	0.030
cg17569154	-0.532	0.437	0.971	0.244	-2.088	0.052	-0.316	0.627	0.083	0.930	0.396	0.024	-1.683	0.200
cg17790129	-1.223	0.308	0.836	0.549	-0.585	0.710	-0.227	0.861	-0.516	0.583	-0.903	0.012	-1.016	0.614
cg19260663	-1.284	0.277	-2.277	0.138	0.862	0.629	-0.822	0.508	-1.116	0.241	-0.288	0.382	2.222	0.350

cg19706795	1.037	0.137	-0.392	0.575	0.300	0.741	0.897	0.210	-0.012	0.984	-0.026	0.850	-1.074	0.299
cg20764575	0.456	0.580	2.810	0.001	-1.000	0.438	-1.236	0.145	-0.812	0.368	0.105	0.508	-0.648	0.714
cg22804000	0.329	0.718	-2.476	0.062	0.568	0.689	0.853	0.480	-3.490	0.001	-0.582	0.049	-1.430	0.483
cg24805239	0.583	0.315	-2.617	0.003	1.681	0.100	-3.444	0.001	-0.769	0.429	0.036	0.891	2.851	0.023
cg25723149	-1.824	0.002	0.977	0.366	-0.993	0.216	-2.305	0.175	1.593	0.484	-4.789	0.040	1.485	0.292
cg26712096	4.227	0.000	1.572	0.029	1.810	0.019	3.199	0.002	0.930	0.012	0.987	0.289	1.683	0.125

Highlighted cells represent significant *p*-values.

Table S5 Table of the linear regression results for the analysis of age and methylation.

**Probe**	**BLCA_slope**	**BLCA_lm_*p*_value**	**BRCA_slope**	**BRCA_lm_*p*_value**	**CRC_slope**	**CRC_lm_*p*_value**	**ESCA_slope**	**ESCA_lm_*p*_value**	**HNSC_slope**	**HNSC_lm_*p*_value**	**KIRC_slope**	**KIRC_lm_*p*_value**	**KIRP_slope**	**KIRP_lm_*p*_value**
cg00473134	3.88E-04	4.49E-01	1.05E-03	5.69E-03	3.79E-03	5.51E-08	3.88E-03	2.75E-03	1.84E-04	1.07E-01	-3.74E-05	5.64E-01	-1.89E-03	2.49E-05
cg01733570	1.20E-03	1.31E-01	7.59E-04	1.33E-01	2.91E-03	5.03E-05	2.96E-03	3.02E-02	1.64E-03	1.32E-03	4.38E-05	9.27E-01	-9.22E-04	2.89E-01
cg03995857	6.49E-04	3.30E-01	1.62E-03	2.41E-03	3.54E-03	9.25E-05	5.40E-03	1.66E-03	2.23E-04	6.48E-02	2.17E-05	7.45E-01	-1.96E-03	3.18E-05
cg04317854	-1.73E-03	4.54E-02	-2.75E-04	5.58E-01	7.09E-04	8.58E-02	-3.01E-04	6.82E-01	-7.67E-05	8.07E-01	1.55E-03	5.72E-03	1.56E-03	3.69E-02
cg04770504	7.40E-04	2.79E-01	1.76E-03	1.26E-04	2.12E-03	3.92E-03	3.54E-03	6.70E-03	-8.93E-05	4.64E-01	5.69E-05	1.62E-01	-1.31E-03	1.16E-04
cg06301139	-9.05E-04	7.06E-02	-1.18E-04	7.49E-01	3.14E-04	5.38E-01	-1.04E-03	8.63E-02	1.24E-03	2.22E-02	-1.09E-04	4.42E-01	7.91E-05	4.29E-01
cg07293520	2.48E-04	6.81E-01	1.31E-03	1.74E-03	4.52E-03	2.27E-07	4.72E-03	3.63E-03	-1.51E-05	9.09E-01	1.02E-05	6.44E-01	-1.80E-03	4.56E-05
cg07320646	1.00E-04	9.03E-01	2.23E-03	1.88E-04	5.14E-03	1.24E-06	5.12E-03	6.90E-03	-3.57E-05	7.95E-01	-1.62E-05	4.08E-01	-1.93E-03	2.35E-04
cg07504598	-1.08E-03	1.33E-01	8.08E-04	9.32E-02	1.87E-03	1.96E-03	2.53E-03	2.55E-02	1.95E-03	1.74E-03	1.28E-04	7.76E-01	-1.80E-03	4.73E-02

cg09333471	6.84E-04	2.51E-01	8.97E-04	2.73E-02	3.10E-03	3.80E-06	3.71E-03	4.54E-03	2.18E-04	4.32E-01	-8.88E-05	6.91E-01	-9.87E-04	4.85E-02
cg12922093	-1.54E-03	8.15E-02	-8.07E-04	1.30E-01	1.17E-04	8.57E-01	-1.93E-03	1.25E-02	-4.62E-04	1.15E-01	-1.58E-04	4.05E-01	5.70E-05	7.56E-01
cg14205998	-5.72E-04	5.04E-01	-6.37E-04	2.15E-01	4.23E-05	9.45E-01	-7.10E-04	3.43E-01	-7.73E-05	6.20E-01	-1.22E-04	1.61E-01	9.17E-06	9.12E-01
cg15037663	-1.81E-03	1.01E-02	-3.06E-04	5.31E-01	1.66E-03	5.11E-03	1.25E-03	2.20E-01	2.22E-03	5.62E-04	-9.67E-05	8.18E-01	-1.54E-03	6.27E-02
cg17569154	2.18E-03	3.02E-02	-8.48E-04	1.05E-01	1.34E-04	8.40E-01	-4.15E-03	2.10E-03	6.43E-04	3.20E-01	3.72E-04	3.58E-01	-7.03E-04	4.45E-01
cg17790129	-4.73E-04	6.15E-01	-1.18E-03	3.88E-02	1.16E-04	8.32E-01	-2.14E-03	1.65E-02	3.88E-04	1.54E-01	1.35E-04	5.45E-01	-2.07E-04	5.43E-01
cg19260663	-4.88E-04	5.92E-01	-9.77E-04	4.84E-02	7.03E-04	1.80E-01	-6.48E-04	3.89E-01	-4.15E-05	8.29E-01	3.18E-04	1.33E-02	1.14E-04	6.24E-01
cg19706795	-1.51E-03	1.06E-02	3.24E-05	9.42E-01	7.27E-04	1.22E-01	4.69E-04	5.92E-01	1.78E-03	4.63E-03	-3.06E-04	4.98E-01	-2.12E-03	1.61E-02
cg20764575	-1.09E-03	5.13E-02	5.78E-04	1.99E-01	8.77E-04	6.30E-03	6.99E-04	4.66E-01	2.37E-03	4.61E-04	1.88E-04	7.45E-01	-2.18E-03	1.58E-02
cg22804000	1.72E-04	8.24E-01	1.27E-03	9.07E-03	2.40E-03	3.83E-04	4.27E-03	1.43E-03	1.43E-03	1.31E-03	6.24E-04	1.58E-01	-1.33E-03	1.10E-01
cg24805239	6.54E-04	3.14E-01	1.86E-03	2.81E-05	3.59E-03	1.31E-05	4.10E-03	5.38E-03	2.34E-04	1.02E-01	1.58E-04	1.34E-01	-1.43E-03	6.31E-04
cg25723149	6.83E-04	4.19E-01	1.88E-03	3.85E-04	3.13E-03	4.49E-06	4.54E-03	2.37E-03	1.55E-03	7.48E-04	8.26E-04	8.71E-02	-1.39E-03	9.97E-02
cg26712096	5.25E-04	5.61E-01	-1.53E-03	5.00E-03	-1.28E-03	7.17E-02	-2.33E-03	3.75E-02	8.06E-04	6.54E-02	-1.56E-05	8.86E-01	1.45E-04	4.10E-01
**Probe**	**LIHC_slope**	**LIHC_lm_*p*_value**	**LUAD_slope**	**LUAD_lm_*p*_value**	**LUSC_slope**	**LUSC_lm_*p*_value**	**PAAD_slope**	**PAAD_lm_*p*_value**	**PRAD_slope**	**PRAD_lm_*p*_value**	**THCA_slope**	**THCA_lm_*p*_value**	**UCEC_slope**	**UCEC_lm_*p*_value**
cg00473134	7.80E-04	1.45E-01	1.52E-04	7.39E-01	3.20E-04	1.44E-01	2.24E-04	7.93E-01	3.71E-03	6.24E-05	1.50E-04	2.30E-02	2.20E-04	6.98E-01
cg01733570	-1.72E-03	2.57E-02	5.90E-04	2.84E-01	-1.84E-03	4.54E-02	2.60E-04	8.14E-01	-3.55E-05	9.49E-01	1.04E-03	3.29E-02	-1.12E-03	2.18E-01
cg03995857	1.24E-03	2.95E-02	1.47E-04	8.14E-01	2.46E-04	3.32E-01	1.24E-04	9.04E-01	3.83E-03	5.62E-05	1.84E-04	9.86E-04	6.48E-05	9.24E-01
cg04317854	-9.43E-04	1.16E-01	1.02E-03	5.00E-02	-1.26E-03	9.04E-02	9.09E-04	1.72E-01	9.42E-04	1.55E-01	6.84E-04	4.78E-02	-1.43E-03	4.07E-02
cg04770504	3.13E-04	2.07E-01	5.64E-04	3.81E-01	2.10E-04	2.26E-01	3.63E-04	6.20E-01	3.06E-03	1.32E-04	9.92E-05	1.14E-03	4.13E-04	4.65E-01

cg06301139	-8.45E-04	1.34E-01	-1.11E-04	7.26E-01	-4.68E-04	4.50E-01	2.07E-04	3.96E-01	-1.10E-04	2.76E-01	4.57E-05	1.25E-01	-6.87E-04	1.12E-01
cg07293520	1.98E-04	1.98E-01	6.85E-04	2.04E-01	3.03E-04	1.28E-01	2.12E-04	6.37E-01	9.60E-04	5.06E-04	3.21E-05	7.67E-02	7.89E-04	1.41E-01
cg07320646	4.59E-04	1.31E-01	6.90E-04	3.26E-01	3.68E-04	9.62E-02	3.57E-04	5.94E-01	5.63E-04	3.51E-02	-1.08E-06	9.54E-01	7.80E-04	2.09E-01
cg07504598	-2.34E-03	2.97E-03	-1.70E-05	9.74E-01	-1.01E-03	2.66E-01	5.02E-04	5.44E-01	7.01E-04	2.88E-01	1.45E-03	2.47E-03	-2.66E-04	7.49E-01
cg09333471	1.36E-03	4.08E-02	2.22E-04	6.56E-01	1.44E-04	7.52E-01	1.28E-03	1.16E-01	2.73E-03	3.80E-03	2.87E-04	2.39E-01	-1.03E-04	8.83E-01
cg12922093	-3.34E-03	2.62E-06	1.63E-04	7.75E-01	-6.40E-04	2.48E-01	1.71E-03	1.68E-02	-1.58E-03	1.46E-02	8.37E-05	4.69E-01	-3.23E-04	4.33E-01
cg14205998	9.27E-05	6.59E-01	2.01E-04	5.85E-01	2.04E-05	9.67E-01	4.80E-05	8.17E-01	-2.40E-04	1.35E-01	-6.59E-05	2.23E-01	1.07E-04	5.74E-01
cg15037663	-3.37E-03	2.71E-05	-1.26E-04	7.96E-01	-8.96E-04	3.46E-01	6.76E-04	3.58E-01	4.89E-05	9.22E-01	1.19E-03	1.08E-02	-1.44E-04	8.64E-01
cg17569154	-1.21E-03	3.65E-02	2.97E-04	5.02E-01	-7.88E-04	2.78E-01	1.06E-03	2.08E-01	-6.23E-04	2.17E-01	-2.43E-05	5.67E-01	1.20E-04	7.81E-01
cg17790129	6.81E-04	1.30E-02	2.00E-04	6.58E-01	-7.85E-04	9.68E-02	-8.27E-04	2.98E-01	1.29E-04	4.22E-01	-1.37E-05	9.12E-01	-1.97E-03	5.55E-04
cg19260663	-3.11E-03	3.33E-05	8.65E-04	8.19E-02	2.86E-04	6.08E-01	8.54E-04	2.21E-01	-2.16E-03	7.24E-03	-1.35E-04	1.41E-01	2.09E-04	6.63E-01
cg19706795	-3.00E-03	8.73E-05	1.37E-04	7.26E-01	-8.23E-04	3.41E-01	-3.66E-05	9.49E-01	-4.53E-04	1.99E-01	5.32E-04	3.55E-02	-2.46E-04	7.64E-01
cg20764575	-3.42E-03	4.92E-05	2.70E-05	9.44E-01	-1.19E-04	8.97E-01	7.56E-04	2.00E-01	-2.72E-04	4.54E-01	2.77E-04	2.82E-01	-1.42E-03	1.31E-01
cg22804000	-1.32E-03	7.73E-02	1.12E-03	3.49E-02	-1.53E-03	6.20E-02	2.93E-04	7.74E-01	1.31E-04	8.58E-01	1.73E-03	1.40E-04	-6.78E-04	4.35E-01
cg24805239	3.52E-04	2.64E-01	9.45E-04	1.09E-01	3.81E-04	1.26E-01	5.95E-04	4.42E-01	3.72E-03	9.18E-05	8.30E-04	4.26E-09	-4.47E-07	9.99E-01
cg25723149	-9.22E-04	2.30E-01	9.39E-04	1.01E-01	-1.15E-03	1.82E-01	3.41E-04	7.46E-01	4.22E-04	5.57E-01	1.61E-03	2.83E-04	-1.06E-03	2.44E-01
cg26712096	-2.00E-03	3.94E-03	3.45E-04	4.97E-01	8.51E-05	9.11E-01	4.06E-04	3.15E-01	-8.35E-04	2.23E-01	-6.21E-05	4.13E-01	8.78E-04	1.54E-01

Highlighted cells represent significant *p*-values.

R packages used:

1 lme4 - Bates D, Mächler M, Bolker B, Walker S. Fitting Linear Mixed-Effects Models using lme4. *J. Stat. Softw*. **2014**, *67*(1), 51. doi:10.18637/jss.v067.i01.

2 Pbkrtest - Halekoh U, Højsgaard S. A Kenward-Roger Approximation and Parametric Bootstrap Methods for Tests in Linear Mixed Models - The R Package pbkrtest. *J. Stat. Softw*. **2014**, *59*(9), 1–32. doi:10.18637/jss.v059.i09.

3 Zhao J.H. Genetic Analysis Package. *J. Stat. Softw*. **2007**, *23*(8), 1–18.doi:http://dx.doi.org/10.18637/jss.v023.i08.

4 MASS–Venables, W.N. (William, N., Ripley, B.D.; Venables, W.N. (William, N.). *Modern Applied Statistics With S*. Vol 45. Fourth. New York: Springer; 2002. doi:10.1198/tech.2003.s33.

5 ROCR–Sing, T.; Sander, O.; Beerenwinkel, N.; Lengauer, T. ROCR: Visualizing classifier performance in R. *Bioinformatics*. 2005, 21(20), 3940–3941. doi:10.1093/bioinformatics/bti623.

6 LeDell, E.; Petersen, M.; van der Laan, M. cvAUC: cross-validated area under the ROC curve confidence intervals. R package version. **2013**: 1-0.

7 Wickham, P.-H. The Split-Apply-Combine Strategy for Data Analysis. *J. Stat. Softw*. **2011**, *40*(1), 1–29. URL http://www.jstatsoft.org/v40/i01/.
